# Mu opioid receptor-mediated release of endolysosome iron increases levels of mitochondrial iron, reactive oxygen species, and cell death

**DOI:** 10.1515/nipt-2022-0013

**Published:** 2023-03-25

**Authors:** Peter W. Halcrow, Nirmal Kumar, Emily Hao, Nabab Khan, Olimpia Meucci, Jonathan D. Geiger

**Affiliations:** Department of Biomedical Sciences, University of North Dakota School of Medicine and Health Sciences, Grand Forks, ND, USA; Department of Physiology and Pharmacology, Drexel University School of Medicine, Philadelphia, PA, USA

**Keywords:** cell death, deferoxamine, endolysosomes, iron, mitochondria, opioids

## Abstract

**Objectives:**

Opioids including morphine and DAMGO activate mu-opioid receptors (MOR), increase intracellular reactive oxygen species (ROS) levels, and induce cell death. Ferrous iron (Fe^2+^) through Fenton-like chemistry increases ROS levels and endolysosomes are “master regulators of iron metabolism” and contain readily-releasable Fe^2+^ stores. However, mechanisms underlying opioid-induced changes in endolysosome iron homeostasis and downstream-signaling events remain unclear.

**Methods:**

We used SH-SY5Y neuroblastoma cells, flow cytometry, and confocal microscopy to measure Fe^2+^ and ROS levels and cell death.

**Results:**

Morphine and DAMGO de-acidified endolysosomes, decreased endolysosome Fe^2+^ levels, increased cytosol and mitochondria Fe^2+^ and ROS levels, depolarized mitochondrial membrane potential, and induced cell death; effects blocked by the nonselective MOR antagonist naloxone and the selective MOR antagonist β-funaltrexamine (β-FNA). Deferoxamine, an endolysosome-iron chelator, inhibited opioid agonist-induced increases in cytosolic and mitochondrial Fe^2+^ and ROS. Opioid-induced efflux of endolysosome Fe^2+^ and subsequent Fe^2+^ accumulation in mitochondria were blocked by the endolysosome-resident two-pore channel inhibitor NED-19 and the mitochondrial permeability transition pore inhibitor TRO.

**Conclusions:**

Opioid agonist-induced increases in cytosolic and mitochondrial Fe^2+^ and ROS as well as cell death appear downstream of endolysosome de-acidification and Fe^2+^ efflux from the endolysosome iron pool that is sufficient to affect other organelles.

## Introduction

Opioids remain one of the most extensively used analgesics clinically and because of their ability to produce euphoria and dependency are subject to abuse. Functioning mainly through opioid receptors (OR), opioid drugs mediate virtually all clinically relevant effects via activation of mu opioid receptors (MOR) [1–4]. Adversely, MOR agonists including morphine and DAMGO ([D-Ala^2^, NMe-Phe^4^, Gly-ol^5^]-enkephalin) can increase levels of reactive oxygen species (ROS) in cytosol and mitochondria, disrupt redox homeostasis, and induce cell death [5–9].

Central to apoptotic cell death is mitochondrial dysfunction and opioid drug-induced cell death is caused by mitochondrial dysfunction [10–12]. Depolarization of the mitochondrial membrane potential (∆ψ_m_) appears to be one of the earliest mitochondrial events leading to cell death [13–16]. Opening of the mitochondrial permeability transition pore (mPTP) depolarizes ∆ψ_m_, releases apoptogenic factors from mitochondria into the cytoplasm, and suppresses mitochondrial oxidative phosphorylation [17–19]. However, even though opioid agonists depolarize ∆ψ_m_, release apoptogenic factors from mitochondria, and suppress mitochondrial oxidative phosphorylation [20–23], the mechanisms by which opioid agonists can cause opioid-induced toxicity remain poorly understood.

A fundamental reactant in ROS generation via Fenton-like chemistry is ferrous iron (Fe^2+^); Fe^2+^ reacts with hydrogen peroxide and oxygen to produce hydroxyl radicals and superoxide anions that damage DNA and proteins [24–26]. Endosomes and lysosomes (endolysosomes) have been termed “master regulators of iron metabolism” central to iron trafficking [27, 28]. However, it was not until recently that concentrations of Fe^2+^ in endolysosomes were determined, that these stores of Fe^2+^ were readily-releasable [29, 30], and were sufficient to affect cytosolic and mitochondrial levels of Fe^2+^ and redox signaling [28, 31, 32]. A defining characteristic of endolysosomes is their acidic lumen and the luminal pH is linked to iron homeostasis; inhibiting endolysosome acidity triggers iron dysregulation [28, 33, 34]. Previously, we reported using U87MG astrocytoma cells and primary cultures of rat cerebral cortical neurons that morphine through activation of MORs and Gαi protein signaling decreased levels of endolysosome Fe^2+^, increased levels of cytosolic Fe^2+^, and led to increased levels of ferritin heavy chain [35, 36]. However, it remains unclear whether opioids working through MORs cause sufficient changes in levels of Fe^2+^ to affect mitochondrial function and cell death.

We have shown previously that dysregulation of endolysosome iron leads to changes in endolysosome heterogeneity including their intracellular position and luminal acidity [29]. Here, we showed using SH-SY5Y neuroblastoma cells that MOR agonists morphine and DAMGO (1) de-acidified endolysosomes, (2) decreased Fe^2+^ levels in endolysosomes, (3) increased levels of Fe^2+^ in cytosol and mitochondria, (4) increased cytosolic and mitochondrial ROS levels, (5) depolarized mitochondrial membrane potential, and (6) induced cell death. Mechanistically, we found that these effects of morphine and DAMGO were blocked by the nonselective MOR antagonist naloxone and by the MOR selective antagonist β-funaltrexamine (β-FNA). In addition, deferoxamine (DFO), the endocytosed specific chelator of endolysosome iron, inhibited opioid agonist-induced increases in cytosolic and mitochondrial Fe^2+^ and ROS. Furthermore, opioid-induced efflux of Fe^2+^ from endolysosomes was blocked by NED-19, an inhibitor of endolysosome-resident two-pore channels (TPC), and opioid-induced accumulation of mitochondrial Fe^2+^ was blocked by the mPTP inhibitor TRO. The MOR antagonists naloxone or β-FNA were able to increase levels of Fe^2+^ in endolysosomes by inhibiting what appeared to be a constitutive leak of Fe^2+^ from endolysosomes. Similar effects were observed with DFO. Thus, opioid agonist-induced increases in cytosolic and mitochondrial Fe^2+^ as well as cell death appear to be downstream of endolysosome de-acidification and the release of Fe^2+^ from endolysosomes. Increased understanding of opioid-induced changes in inter-organellar signaling may yield important new insight into the pharmacological actions of and toxic reactions to opioid analgesics.

## Materials and methods

The experiments performed in this manuscript were not preregistered. No blinding procedures were performed. No statistical method was employed to pre-determine the sample size of the experiments. No randomization methods were used.

**Cell cultures:** Human neuroblastoma (SH-SY5Y) cells were cultured in Dulbecco’s Modified Eagle Medium (Invitrogen, cat. no. 11995, Carlsbad, USA) containing 10% fetal bovine serum (ThermoFisher, Waltham, USA) and 1% penicillin/streptomycin (Invitrogen, cat. no. 15140122, Carlsbad, USA). SH-SY5Y cells were grown in T75 flasks and subcultured as needed to 70–80% confluency. Cells were passaged every 3–4 days using 0.025% trypsin (Invitrogen, Carlsbad, USA) and maintained in an incubator set at 37 °C and 5% CO_2_. Cells were not used past their tenth passage. The cells used in this manuscript are not listed as a commonly misidentified cell line by the International Cell Line Authentication Committee and no authentication experiments were conducted.

**Endolysosome pH:** Endolysosome pH was determined using LysoSensor Yellow/Blue DND-160 (ThermoFisher, cat. no. L7545, Waltham, USA), a ratiometric dual-excitation dye used to measure pH in acidic organelles independent of dye loading efficiency. SH-SY5Y cells were grown in 35 mm^2^ dishes overnight and then incubated for 5 min at 37 °C with media containing 10 µM DND-160. After incubation, the media was replaced with new media and then images were taken. During imaging, various drug treatments were added. Light emission at a wavelength of 520 nm in response to excitation for 2 ms at 340/380 nm was measured every 30 s using a filter-based microscope imaging system (Zeiss, Germany). Using a standard curve we calculated the pH as previously described [37] and we calculated changes in proton concentrations within endolysosomes using the formula pH = −log[H^+^].

**Endolysosome iron:** Labile ferrous iron (Fe^2+^) in endolysosomes was detected using the fluorescence probe FeRhoNox-1 (Goryo Chemicals, cat no. GC901, Darmstadt, Germany) as described recently by us [29]. SH-SY5Y cells were added to 35 mm^2^ dishes at a density of 7 × 10^4^ cells/dish and incubated overnight prior to being taken for experimentation. Cells were incubated with 10 µM FeRhoNox-1 at 37 °C for 1 h, stained with LysoTracker^TM^ Green DND-26 (Invitrogen, cat. no. L7526, Carlsbad, USA) for 10 min, washed three times with warm PBS, and then imaged using confocal scanning microscopy (Zeiss, LSM800). Imaging settings remained the same throughout each set of experiments; images were acquired just prior to (0 h) and 30 min after treatments were applied. Cells were preincubated with MOR antagonists and other blockers for 30 min prior to the various drug treatments such as morphine and DAMGO. Imaris software version 9.7 (Oxford Instruments, Concord, USA) was used to reconstruct the images and data were represented as mean fluorescence intensity (MFI).

**Cytosolic iron:** Cytosolic Fe^2+^ levels were determined with Phen Green^TM^ SK, diacetate (PGSK) (Invitrogen, cat. no. P14313, Carlsbad, USA). SH-SY5Y cells cultured in 12-well plates were preincubated with MOR antagonists and other blockers for 30 min, incubated for 1 h with various treatments, washed two times with PBS, and incubated at 37 °C and 5% CO_2_ for 20 min with 10 µM PGSK. Cells were then washed with PBS to remove unloaded dye and fluorescence was measured at an excitation of 488 nm and an emission of 530 nm using a flow cytometer (Attune NxT, ThermoFisher, Waltham, USA). Unstained cells were used as controls for background fluorescence and positive controls included cells treated with 10 µM of the weak-base drug chloroquine (CQ). Mean fluorescence intensity (MFI) data were collected from a minimum of 10,000 cells per condition.

**Cytosolic ROS:** Levels of cytosolic ROS were measured using approximately 6 × 10^5^ SH-SY5Y cells/well following preincubation with MOR antagonists and other blockers for 30 min followed by 1 h incubations with various treatments. Following the incubations, cells were washed three times with PBS, 10 µM CM-H_2_DCFDA (Invitrogen, cat. no. C6827, Carlsbad, USA) was added, and cells were incubated for 30 min at 37 °C and 5% CO_2_; cells were then washed three times with PBS to remove extracellular stain. Unstained cells were used as negative controls to establish background fluorescence and stained cells incubated with 250 µM H_2_O_2_ were used as positive controls. Mean fluorescence intensity (MFI) was acquired on a minimum of 10,000 cells per condition using an Attune NxT flow cytometer (ThermoFisher, Waltham, USA).

**Mitochondrial iron:** Mitochondrial Fe^2+^ levels were measured using rhodamine B 4-[(2,2′-bipyridin-4-yl) aminocarbonyl] benzyl ester (RDA) (Guidechem, cat. no. 952228-30-7, Milwaukee, USA). SH-SY5Y cells were added at a density of about 6 × 10^5^ cells/well to 12-well plates and incubated at 37 °C and 5% CO_2_ overnight prior to preincubation with MOR antagonists and other blockers for 30 min and then various treatments for 1 h. Following treatments, cells were washed three times with PBS and incubated with 100 nM RDA for 20 min in PBS in a 37 °C and 5% CO_2_ incubator_._ Cells were washed with PBS to remove extracellular dye. A minimum of 10,000 cells were collected and RDA fluorescence was analyzed at an excitation of 562 nm and an emission of 598 nm. Unstained cells were used as negative controls and cells treated with FeCl_3_ were used as positive controls. Mean fluorescence intensity (MFI) was determined by flow cytometry (Attune NxT, ThermoFisher, Waltham, USA).

**Mitochondrial ROS:** SH-SY5Y cells (6 × 10^5^ cells/well) were added into 12-well plates and incubated at 37 °C and 5% CO_2_ overnight prior to being taken for experimentation. Cells were preincubated with MOR antagonists and other blockers for 30 min, then cells were treated with respective drugs for 1 h, washed gently three times with PBS, and incubated with 2.5 µM of MitoSox (Invitrogen, cat. no. M36008, Carlsbad, USA) for 20 min in a 37 °C and 5% CO_2_ incubator. Cells were rinsed three times with PBS, fresh PBS was added to the wells, and a minimum of 10,000 cells per condition were placed into Eppendorf tubes for determination of MitoSox fluorescence at an excitation of 510 nm and an emission of 580 nm by flow cytometry (Attune NxT, ThermoFisher, Waltham, USA).

**Mitochondrial membrane potential:** SH-SY5Y cells were added to 12-well plates at a density of 6 × 10^5^ cells/well and were incubated overnight at 37 °C and 5% CO_2_. Cells were preincubated with MOR antagonists and other blockers for 30 min, then treated with respective drugs for 1 h, washed gently three times, and treated with 100 nM MitoTracker™ Red CMXROS (Invitrogen, cat. no. M7512, Carlsbad, USA) for 30 min in a 37 °C and 5% CO_2_ incubator. Cells were washed three times with warm PBS, re-suspended in 1 mL PBS, and fluorescence was determined using a flow cytometer (Attune NxT, ThermoFisher, Waltham, USA) at an excitation of 579 nm and an emission of 599 nm. For confocal microscopy, SH-SY5Y cells were added to 35 mm^2^ dishes and incubated overnight in an incubator kept at 37 °C and 5% CO_2_. Cells were treated with respective drugs for 1 h, washed gently three times with PBS, and incubated with 100 nM Mito-Tracker Red CMXROS and 200 nM MitoTracker^TM^ Green FM (Invitrogen, cat. no. M7514, Carlsbad, USA) for 30 min in a 37 °C and 5% CO_2_ incubator. Cells were washed three times with PBS, stained with 1 μg/mL Hoechst 33342 for 10 min, washed with PBS three times, and imaged using confocal microscopy (Zeiss 8000, Jena, Germany) at an excitation of 579 nm and an emission of 599 nm.

**Cell death:** Cell viability was determined using propidium iodide (PI) (BD Biosciences, cat. no. 556463, Franklin Lakes, USA) and flow cytometry. SH-SY5Y cells were added to 12-well plates at a density of about 60,000 cells/well, incubated overnight at 37 °C and 5% CO_2_, and cells were treated with respective drugs for 24 h. MOR antagonists, DFO, NED-19, and TRO were added to the cells 30 min prior to adding morphine and DAMGO. After 24 h, cells were stained with PI (3 µM) at room temperature for 15 min, and fluorescence was determined using a flow cytometer (Attune NxT, ThermoFisher, Waltham, USA). For determination of apoptotic and necrotic cell death, Apoptosis/Necrosis Detection kits (Abcam, cat no. ab176749, Cambridge, UK) were used. This method simultaneously determines numbers of apoptotic, necrotic and live cells; 7-AAD labels the nucleus of necrotic cells (red fluorescence), Apopxin Green labels phosphatidylserine (PS) on the cell surface of apoptotic cells (green fluorescence), and CytoCalcein Violet labels the cytoplasm of living cells (blue fluorescence). SH-SY5Y cells were seeded into 35 mm^2^ glass-bottom dishes at a density of about 1 × 10^4^ cells and incubated overnight at 37 °C and 5% CO_2_. Cells were treated with respective drugs for 24 h and then incubated with a mixture of 200 µL assay buffer, 2 µL Apopxin Green Indicator, 1 µL CytoCalcein 450, and 1 µL 7-AAD at room temperature for 45 min in the dark. For determination of ferroptotic cell death, treated SH-SY5Y cells were stained with 10 µM of C11-BODIPY 581/591 (Invitrogen, cat. no. D3861, Carlsbad, USA) for 30 min at 37 °C as a measure of lipid peroxidation. Cells were washed two times with PBS and images were acquired using a spinning disk confocal laser microscope (Oxford Instruments, Concord, USA).

**Reagents:** Chemical reagents were obtained from ThermoFisher Scientific except as follows; TRO-19622 (TOCRIS, cat. no. 2906, Bristol, UK), naloxone hydrochloride (TOCRIS, cat. no. 0599, Bristol, UK), NED-19 (TOCRIS, cat. no. 3954, Bristol, UK), morphine sulfate (Sigma-Aldrich, cat. no. M8777, St. Louis, USA), β-funaltrexamine hydrochloride (Sigma-Aldrich, cat. no. 0003, St. Louis, USA), DAMGO (Sigma-Aldrich, cat. no. E7384, St. Louis, USA), auranofin (TOCRIS, cat. no. 4600, Bristol, UK), nicotinic acid adenine dinucleotide phosphate tetrasodium salt (TOCRIS, cat. no. 3905, Bristol, UK), and bafilomycin A1 (TOCRIS, cat. no. 1334, Bristol, UK).

**Statistical analyses:** All data were reported as means ± standard deviation (SD). Statistical significance between controls and treatments (two groups) were analyzed with a two-tailed Student’s *t*-test, and statistical significance between 3 groups or more (multiple groups) were analyzed with one-way ANOVA plus a Tukey’s multiple comparison post-hoc test. A p-value less than 0.05 was considered to be statistically significant. GraphPad Prism 9.3.1 software was used to perform all statistical analyses. No pre-determined exclusion criteria were performed. Normal distribution of data was assessed for statistical analysis by using the Shapiro-Wilk test (p<0.05 was considered as not to conform to a normal distribution). The Kruskal–Wallis non-parametric test was used to compare data that had a skewed distribution. The number of cells used for this study was determined based on previous studies [38–40]. No outliers were detected using ROUT method. Results were based on experiments conducted at least three separate times; each time in triplicate.

**Ethical statement:** Institutional ethical approval was not required for this study.

## Results

**Figure 1:**
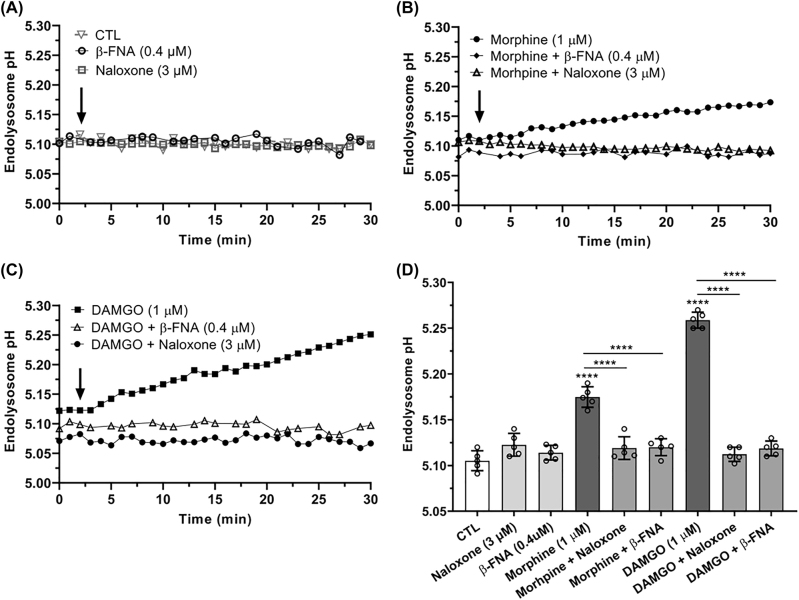
Morphine- and DAMGO-induced endolysosome de-acidification was blocked by the MOR antagonists naloxone and β-FNA. (A) Treatment of SH-SY5Y cells with naloxone (3 µM) or β-FNA (0.4 µM) did not significantly affect endolysosome pH. (B, C) Treatment with morphine (1 µM) and DAMGO (1 µM) significantly (p<0.0001) de-acidified endolysosomes and the MOR antagonists naloxone (3 µM) and β-FNA (0.4 µM) significantly blocked these MOR agonist-induced changes to endolysosome pH. (D) A quantitative bar graph demonstrating peak responses of endolysosome pH over a 30 min timeframe. Individual data points as well as mean ± SD values were included on each bar. An ANOVA with Tukey’s post hoc multiple comparisons test was used for analysis. Each data point represents the mean pH measurement of 50 endolysosomes from five cells performed independently from five cell-culture preparations for each group (n=250). n=the total number of endolysosomes per group. Cells were chosen randomly in the microscope’s field of view with 25 cells used for experimentation per group and no cells were intentionally excluded. (D) F(8, 36)=122.1, p<0.0001.

**Morphine- and DAMGO-induced endolysosome de-acidification was blocked by naloxone and β-FNA:** Because endolysosome de-acidification is known to affect the concentration of Fe^2+^ in endolysosomes ([Fe^2+^]_el_), we first determined the effects of MOR agonists on endolysosome pH. The MOR agonists morphine (1 µM) and DAMGO (1 µM) significantly (p<0.0001) de-acidified endolysosomes (Figure 1A–D). Endolysosome de-acidification by morphine (Figure 1B) and DAMGO (Figure 1C) increased slowly to peak effects observed at 30 min and remained elevated for at least an additional period of 30 min. Pre-treatment of cells for 30 min with the MOR antagonists naloxone (3 µM) and β-FNA (0.4 µM) significantly blocked morphine- (p<0.001) and DAMGO- (p<0.0001) induced endolysosome de-acidification (Figure 1). The MOR antagonists naloxone and β-FNA alone did not significantly affect endolysosome pH (Figure 1A, D). MOR agonist-induced changes in pH corresponded to intraluminal proton concentration changes of 15% for morphine and 28% for DAMGO.

**Figure 2:**
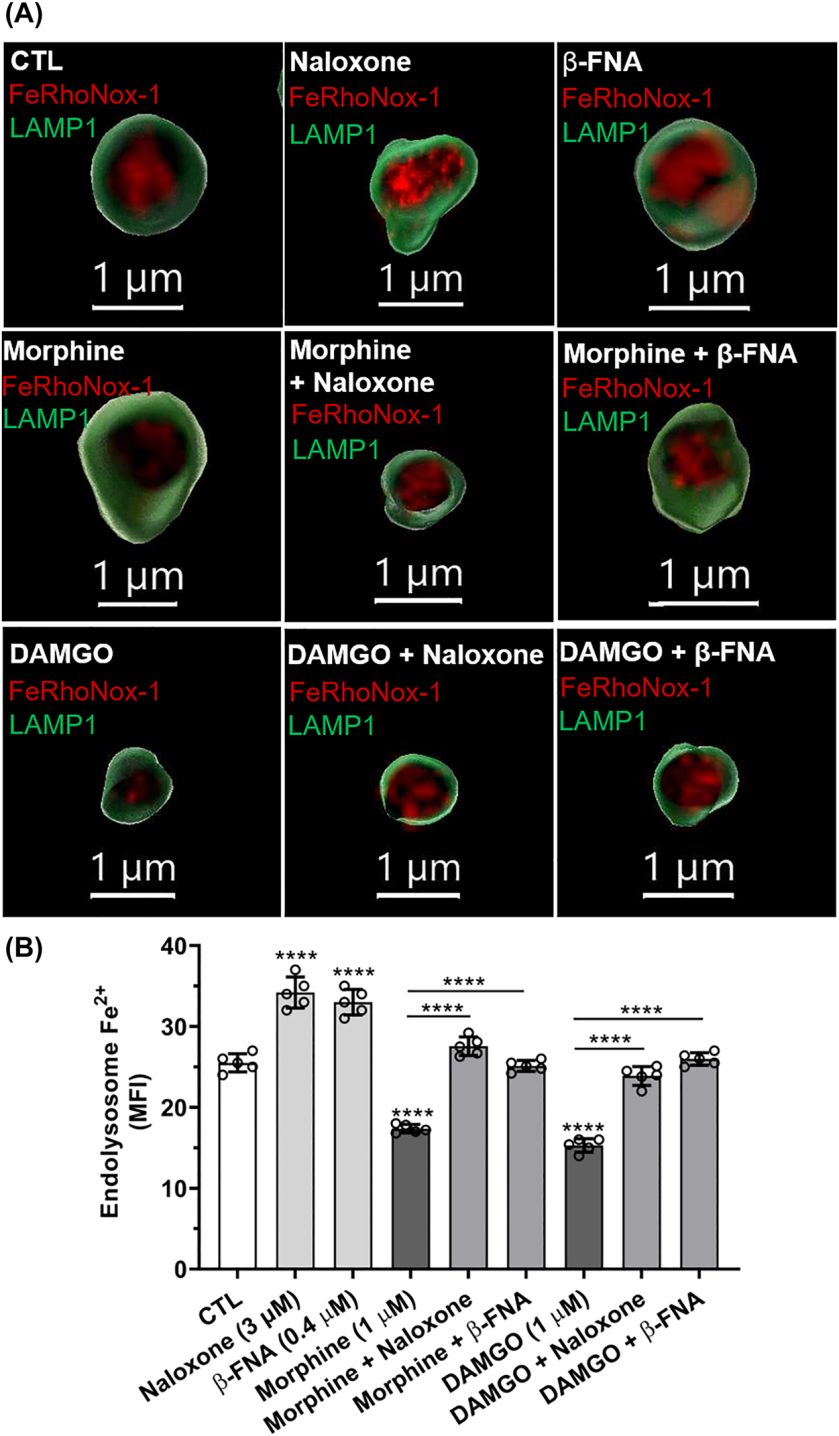
Morphine- and DAMGO-induced decreases in levels of endolysosome Fe^2+^ were blocked by the MOR antagonists naloxone and β-FNA. (A) Qualitatively, the MOR agonists morphine and DAMGO decreased FeRhoNox-1 staining of endolysosome Fe^2+^ and these decreases were blocked by the MOR antagonists naloxone and β-FNA. Images were obtained using confocal scanning microscopy of SH-SY5Y cells treated for 30 min with water (CTL), morphine (1 µM), DAMGO (1 µM), naloxone (3 µM), and β-FNA (0.4 µM). Images were reconstructed using Imaris 3D software and show FeRhoNox-1 staining (red) of Fe^2+^ inside of LAMP1-positive (green) endolysosomes. (B) Quantitatively, levels of endolysosome Fe^2+^ as indicated by mean fluorescence intensity (MFI) for FeRhoNox-1 staining were significantly (p<0.0001) decreased by morphine (1 µM) and DAMGO (1 µM); the decreases in FeRhoNox-1 staining were blocked by the MOR antagonists naloxone (3 µM) and β-FNA (0.4 µM). An ANOVA with Tukey’s post hoc multiple comparisons test was used for analysis. Each data point represents the mean fluorescence of 300 endolysosomes from ten cells performed independently from three cell-culture preparations for each group (n=9000). n=total number of endolysosomes for each group plotted. Cells were chosen randomly in the microscope’s field of view with 30 cells used for experimentation per group and no cells were intentionally excluded. Scale bar=1 µm. (B) F(8, 36)=143.2, p<0.0001.

**Morphine- and DAMGO-induced decreases in [Fe^2+^]_el_ were blocked by naloxone and β-FNA:** Endolysosome de-acidification can induce the efflux of divalent cations from endolysosomes [41–43]. Accordingly, we next determined the extent to which the MOR agonists morphine and DAMGO affected [Fe^2+^]_el_. Using methods we described previously [29, 44], morphine (1 µM) and DAMGO (1 µM) decreased FeRhoNox-1 fluorescence staining for Fe^2+^; effects that were blocked with the MOR antagonists naloxone (3 µM) and β-FNA (0.4 µM) (Figure 2A). Images of single organelles (Figure 2A) demonstrate the mean FeRhoNox-1 fluorescence intensities within endolysosomes per treatment; values were derived from imaging fields of multiple cells (Supplemental Figure 1). Quantitatively, morphine and DAMGO significantly (p<0.0001) decreased [Fe^2+^]_el_ by 32 and 40%, respectively (Figure 2B). Pre-treatment of cells with naloxone and β-FNA blocked significantly (p<0.0001) morphine- and DAMGO-induced decreases in [Fe^2+^]_el_ (Figure 2B). MOR antagonists naloxone (3 µM) and β-FNA (0.4 µM) alone significantly increased [Fe^2+^]_el_ (Figure 2A, B). However, at lower concentrations naloxone (1 µM) and β-FNA (0.1 µM) did not significantly affect [Fe^2+^]_el_ (Supplemental Figure 2), but still significantly decreased morphine- and DAMGO-induced decreases in [Fe^2+^]_el_ (data not shown).

**Figure 3:**
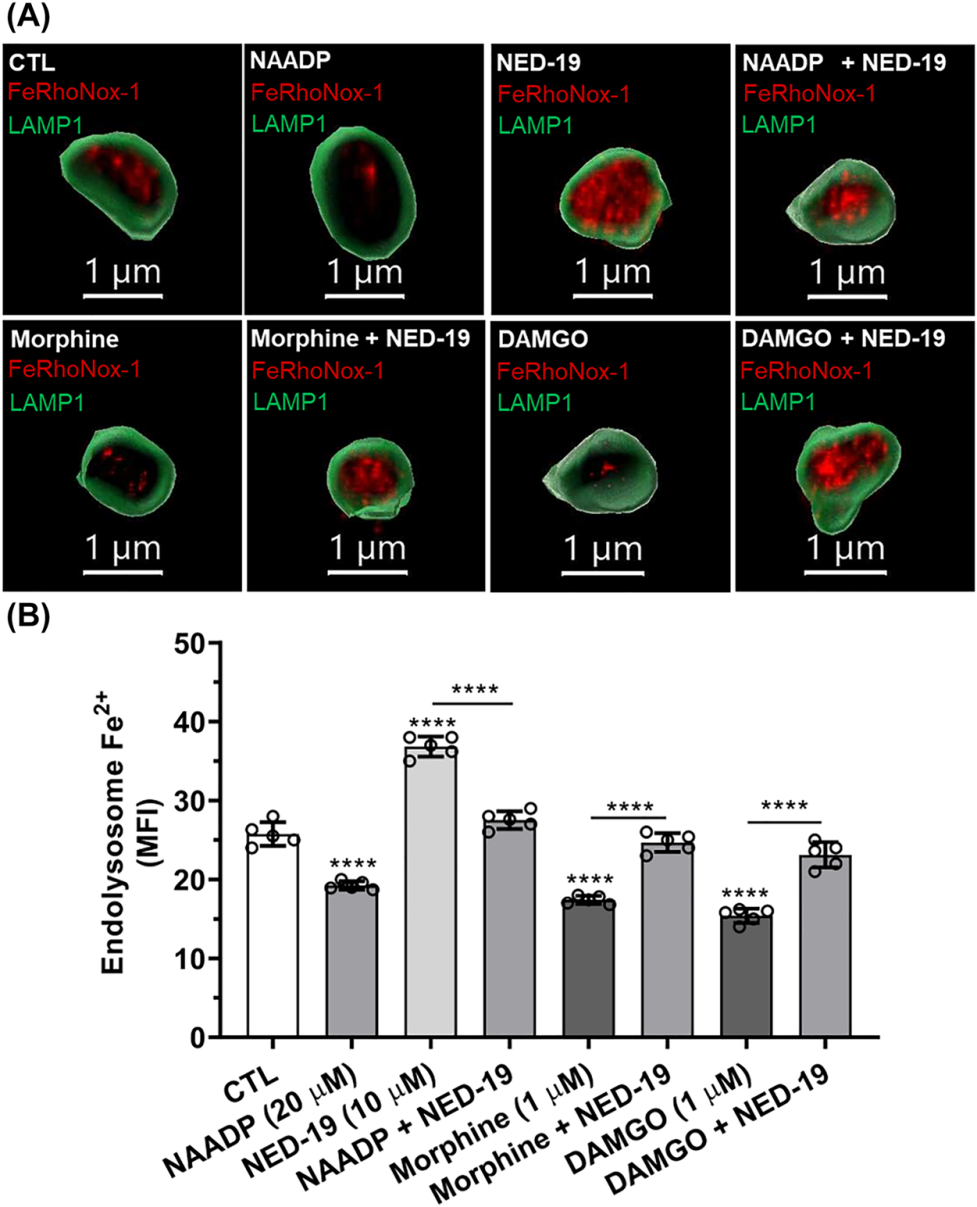
Morphine- and DAMGO-induced decreases in levels of endolysosome Fe^2+^ were blocked by NED-19, an inhibitor of two-pore channels. (A) Qualitatively, activation of two-pore channels with the endogenous agonist NAADP-AM (20 µM) and the MOR agonists morphine (1 µM) and DAMGO (1 µM) decreased FeRhoNox-1 staining of endolysosome Fe^2+^. The effects of NAADP-AM, morphine and DAMGO were blocked by the two-pore channel blocker NED-19 (10 µM). Images were obtained using confocal scanning microscopy of SH-SY5Y cells treated for 30 min with water or DMSO (CTL), morphine (1 µM), DAMGO (1 µM), NAADP-AM (20 µM) and NED-19 (10 µM). Images were reconstructed using Imaris 3D software and show FeRhoNox-1 staining (red) of Fe^2+^ inside of LAMP1-positive (green) endolysosomes. (B) Quantitatively, levels of endolysosome Fe^2+^ as indicated by mean fluorescence intensity (MFI) for FeRhoNox-1 staining were significantly (p<0.0001) decreased by morphine (1 µM), DAMGO (1 µM) and NAADP-AM (20 µM). The decreases in FeRhoNox-1 staining were blocked by the two-pore antagonist NED-19 (10 µM). An ANOVA with Tukey’s post hoc multiple comparisons test was used for analysis. Each data point represents the mean fluorescence of 300 endolysosomes from ten cells performed independently from three cell-culture preparations for each group (n=9000). n=total number of endolysosomes for each group plotted. Cells were chosen randomly in the microscope’s field of view with 30 cells used for experimentation per group and no cells were intentionally excluded. Scale bar=1 µm. (B) F(7, 32)=174.8, p<0.0001.

**Morphine- and DAMGO-induced decreases in levels of endolysosome Fe^2+^ were blocked by NED-19, an inhibitor of two-pore channels:** We next determined the extent to which the release of endolysosome Fe^2+^ induced by morphine and DAMGO involved endolysosome-resident two-pore channels (TPCs) because of their known ability to transport Fe^2+^ [30, 45, 46]. Nicotinic acid adenine dinucleotide phosphate acetoxymethyl ester (NAADP-AM, 20 µM), a TPC activator, significantly decreased [Fe^2+^]_el_ by 22% (Figure 3A, B). NED-19 (10 µM), an antagonist of TPCs, increased significantly (p<0.0001) [Fe^2+^]_el_ by 48% and pre-treatment with NED-19 blocked significantly (p<0.0001) NAADP-induced decreases in [Fe^2+^]_el_ (Figure 3A, B). As above, morphine and DAMGO significantly (p<0.0001) decreased [Fe^2+^]_el_ (Figure 3A, B) and pre-treatment of cells with NED-19 blocked significantly (p<0.0001) morphine- and DAMGO-induced decreases in [Fe^2+^]_el_ (Figure 3A, B). Images of single organelles (Figure 3A) demonstrate the mean FeRhoNox-1 fluorescence intensities within endolysosomes per treatment; values were derived from imaging fields of multiple cells (Supplemental Figure 3).

**Figure 4:**
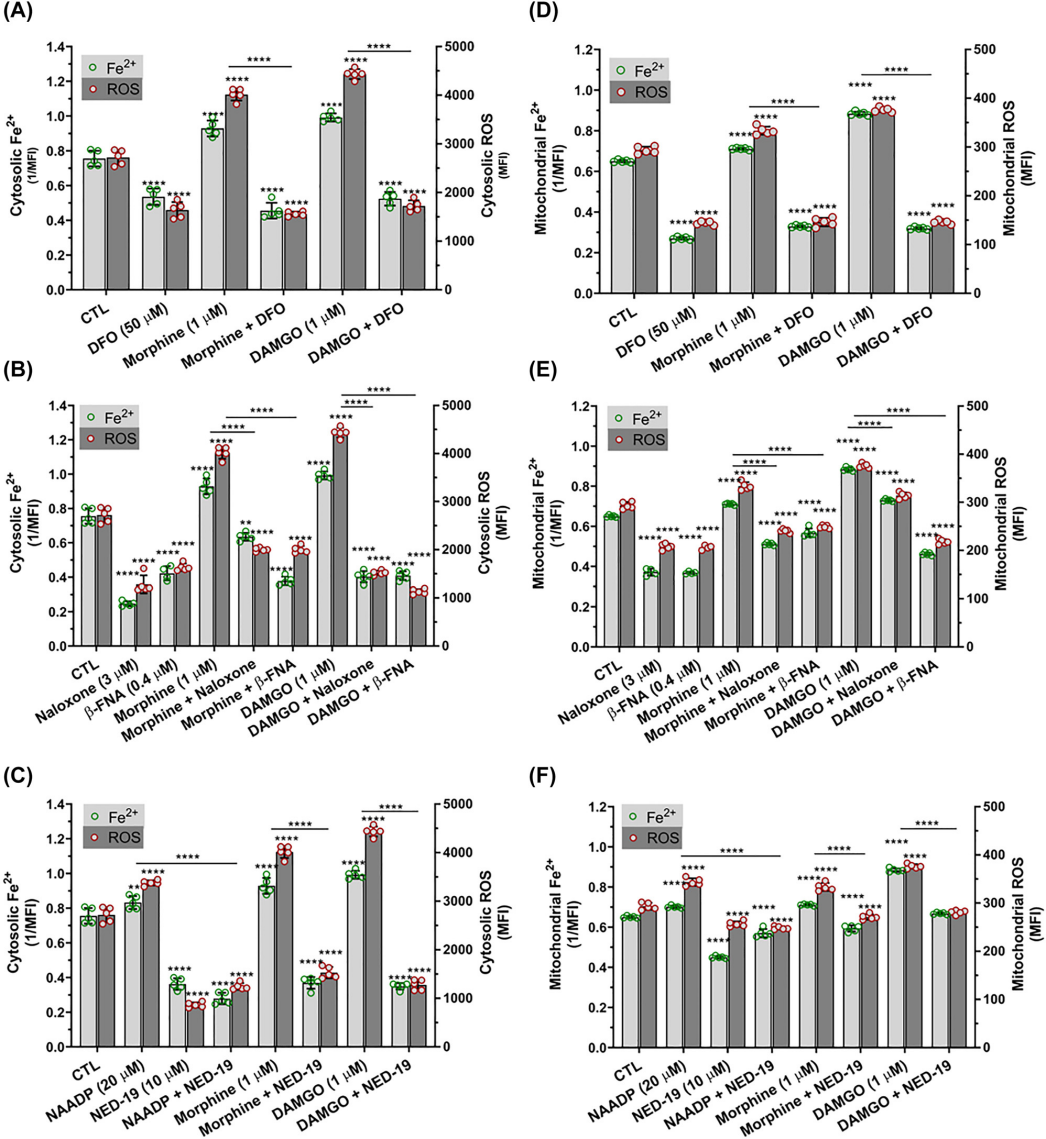
Effects of endolysosome stores of Fe^2+^, MOR activation and two-pore channels on cytosolic and mitochondrial levels of Fe^2+^ and ROS. (A–C) Levels of cytosolic Fe^2+^ (solid bars) were measured with the quenching dye PhenGreen SK (PGSK) and data were transformed and illustrated as the reciprocal of mean fluorescence intensity (1/MFI). Levels of cytosolic ROS (hatched bars), including hydroxyl radicals, were measured using 5- and 6-chloromethyl-2’,7’-dichlorodihydrofluorescein diacetate acetyl ester (CM-H_2_DCFDA). (A) Pre-treatment of cells for 1 h with the endolysosome-specific iron chelator deferoxamine (DFO, 50 µM) significantly decreased levels of cytosolic Fe^2+^ and ROS and significantly decreased morphine- and DAMGO-induced increases in levels of cytosolic Fe^2+^ and ROS. (B) MOR agonists morphine (1 µM) and DAMGO (1 µM) significantly increased levels of cytosolic Fe^2+^ and ROS; these increases were blocked significantly by the MOR antagonists naloxone (3 µM) and β-FNA (0.4 µM). (c) NAADP-AM (20 µM) activation of endolysosome two-pore channels and MOR agonists morphine (1 µM) and DAMGO (1 µM) significantly increased levels of cytosolic Fe^2+^ and ROS; pre-treatment of cells with an inhibitor of two-pore channels NED-19 (10 µM) significantly blocked the effects of NAADP-AM, morphine and DAMGO. (D)–(F) Mitochondrial iron levels ([Fe^2+^]_mito_) were determined by using the quenching dye rhodamine B-[(2,2′-bipyridine-4-yl)-aminocarbonyl]benzyl ester (RDA) and fluorescence data expressed as 1/MFI. Levels of mitochondrial ROS ([ROS]_mito_) were measured using MitoSox and data were expressed as MFI. (D) Pre-treatment of cells for 1 h with the endolysosome-specific iron chelator deferoxamine (DFO, 50 µM) significantly decreased levels of mitochondrial Fe^2+^ and ROS and significantly decreased morphine- and DAMGO-induced increases in mitochondrial Fe^2+^ and ROS. (E) MOR agonists morphine (1 µM) and DAMGO (1 µM) significantly increased levels of mitochondrial Fe^2+^ and ROS; these increases were blocked significantly by the MOR antagonists naloxone (3 µM) and β-FNA (0.4 µM). (F) NAADP-AM (20 µM) activation of endolysosome two-pore channels and MOR agonists morphine (1 µM) and DAMGO (1 µM) significantly increased levels of mitochondrial Fe^2+^ and ROS, and pre-treatment of cells with an inhibitor of two-pore channels NED-19 (10 µM) significantly blocked the effects of NAADP-AM, morphine and DAMGO. An ANOVA with Tukey’s post hoc multiple comparisons test was used for analysis. Each data point represents the mean fluorescence from 10,000 cells performed independently from five cell-culture preparations for each group (n=50,000). n=total number of cells for each group plotted. 50,000 cells were used for experimentation per group and no cells were intentionally excluded. (A) F(5, 48)=527.8, p<0.0001; (B) F(8, 71)=609.3, p<0.0001; (C) F(7, 64)=p<0.0001; (D) F(5, 48)=1408, p<0.0001; (E) F(8, 69)=528, p<0.0001; (F) F(7, 64)=280.1, p<0.0001.

**Effects of endolysosome stores of Fe^2+^, MOR activation and TPCs on cytosolic and mitochondrial levels of Fe^2+^ and ROS:** We next investigated the extent to which and mechanisms by which morphine and DAMGO increased levels of cytosolic Fe^2+^ ([Fe^2+^]_cyt_) and ROS (Figure 4A–C) as well as levels of mitochondrial Fe^2+^ ([Fe^2+^]_mito_) and ROS (Figure 4D–F). Morphine (1 µM) and DAMGO (1 µM) significantly (p<0.0001) increased [Fe^2+^]_cyt_ and [Fe^2+^]_mito_ and significantly (p<0.0001) increased levels of cytosolic and mitochondrial ROS (Figure 4A, D). DFO (50 µM) alone significantly (p<0.0001) decreased [Fe^2+^]_cyt_ and [Fe^2+^]_mito_ and significantly (p<0.0001) decreased cytosolic and mitochondrial levels of ROS; pre-treatment with DFO significantly (p<0.0001) blocked morphine- and DAMGO-induced increases in [Fe^2+^]_cyt_ and [Fe^2+^]_mito_ as well as increases in cytosolic and mitochondrial ROS (Figure 4A, D). At higher concentrations, naloxone (3 µM) and β-FNA (0.4 µM) significantly decreased [Fe^2+^]_cyt_ and [Fe^2+^]_mito_ as well as cytosolic and mitochondrial ROS levels compared to controls (Figure 4B, E). Pre-treatment with naloxone (3 µM) and β-FNA (0.4 µM) significantly (p<0.0001) blocked morphine- and DAMGO-induced increases in [Fe^2+^]_cyt_ and [Fe^2+^]_mito_ as well as cytosolic and mitochondrial ROS (Figure 4B, E). At lower concentrations, naloxone (1 µM) and β-FNA (0.1 µM) had no significant effect on [Fe^2+^]_mito_ and mitochondrial ROS levels (Supplementary Figure 4), but still significantly blocked morphine- and DAMGO-induced increases in [Fe^2+^]_cyt_ and [Fe^2+^]_mito_ as well as cytosolic and mitochondrial ROS. The TPC antagonist NED-19 (10 µM) significantly decreased [Fe^2+^]_cyt_ and [Fe^2+^]_mito_ and significantly (p<0.0001) blocked NAADP- as well as morphine- and DAMGO-induced increases in [Fe^2+^]_cyt_ and [Fe^2+^]_mito_ (Figure 4C, F). Similarly, NED-19 significantly (p<0.0001) decreased cytosolic and mitochondrial ROS and significantly (p<0.0001) blocked NAADP-, morphine- and DAMGO-induced increases in cytosolic and mitochondrial ROS (Figure 4C, F).

**Figure 5:**
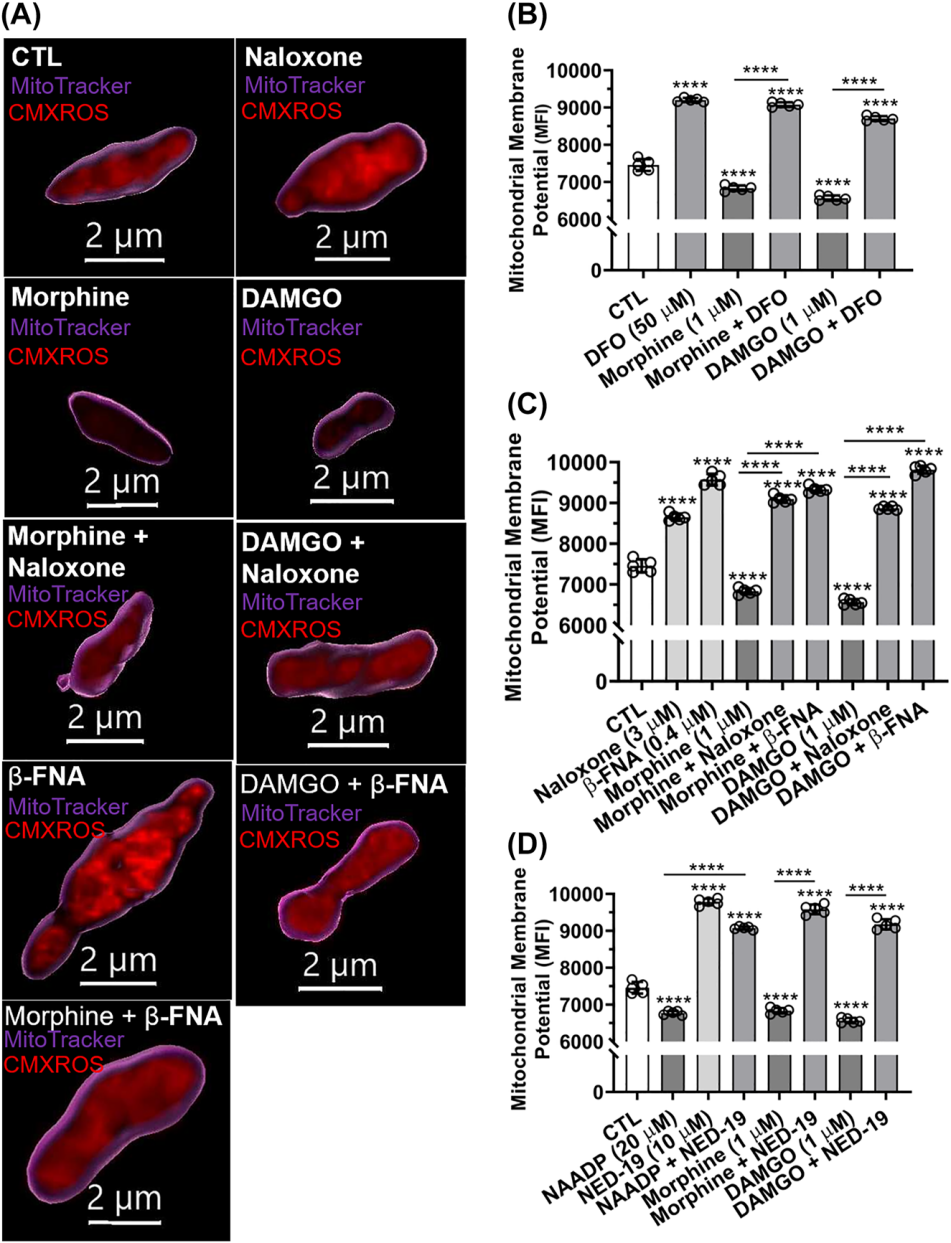
Effects of MOR agonists and antagonists, deferoxamine, and two-pore channel activator and blocker on mitochondrial membrane potential. Mitochondrial membrane potential (∆ψ_m_) was measured using the dye CMXROS; dye intensity decreases with ∆ψ_m_ depolarization. (A) Representative confocal scanning microscope images of mitochondria in SH-SY5Y cells treated for 30 min with water (CTL), and the MOR agonists morphine (1 µM) and DAMGO (1 µM) in the absence or presence of MOR antagonists naloxone (3 µM) and β-FNA (0.4 µM). Images were reconstructed using Imaris 3D software; illustrated are MitoTracker-(purple) positive mitochondria containing CMXROS stain (red). Qualitatively, treatment of cells for 30 min with morphine (1 µM) and DAMGO (1 µM) decreased CMXROS fluorescence, and naloxone and β-FNA inhibited morphine- and DAMGO-induced decreases in CMXROS fluorescence. (B) Quantitatively, morphine- (1 µM) and DAMGO- (1 µM) induced significant decreases in CMXROS mean fluorescence intensity (depolarization) which were blocked by 30 min pre-treatment with DFO (50 µM); DFO alone significantly increased levels of CMXROS. (C) Morphine (1 µM) and DAMGO (1 µM) significantly decreased CMXROS mean fluorescence intensity (depolarization); 30 min pre-treatment with naloxone (1.0 µM) or β-FNA (0.4 µM) followed by treatment with morphine (1 µM) or DAMGO (1 µM) resulted in significantly increased levels of CMXROS (polarization). (D) NAADP-AM (20 µM), morphine (1 µM) and DAMGO (1 µM) decreased ∆ψ_m_; these effects were blocked by 30 min pre-treatments with NED-19 (10 µM) and NED-19 alone significantly increased levels of CMXROS. An ANOVA with Tukey’s post hoc multiple comparisons test was used for analysis. Each data point represents the mean fluorescence from 10,000 cells performed independently from five cell-culture preparations for each group (n=50,000). n=total number of cells for each group plotted. 50,000 cells were used for experimentation per group and no cells were intentionally excluded. (B) F(5, 24)=875.7, p<0.0001; (C) F(8, 36)=745.2, p<0.0001; (D) F(7, 32)=892.2, p<0.0001.

**Morphine- and DAMGO-induced mitochondrial membrane potential depolarization was blocked by DFO, MOR antagonists, and the TPC blocker NED-19:** We next determined the effects of endolysosome Fe^2+^ chelation, agonists and antagonists of MOR, and activators and inhibitors of TPCs on mitochondrial function as indicated by mitochondrial membrane potential (∆ψ_m_). Qualitatively, treatment of SH-SY5Y cells for 1 h with the MOR agonists morphine (1 µM) and DAMGO (1 µM) decreased CMXROS fluorescence staining (depolarization of ∆ψ_m_) and these effects were blocked by the MOR antagonists naloxone and β-FNA (Figure 5A). Morphologically, naloxone and β-FNA both caused enlargement of mitochondria (Figure 5A). Images of single organelles (Figure 5A) demonstrate the mean CMXROS fluorescence intensities within mitochondria per treatment; values were obtained from imaging fields of multiple cells (Supplemental Figure 5). Cells pre-treated for 1 h with deferoxamine (DFO, 50 µM) significantly (p<0.0001) blocked morphine- and DAMGO-induced depolarization of ∆ψ_m_; DFO alone significantly (p<0.0001) increased (polarization) ∆ψ_m_ (Figure 5B). Pre-treatment with naloxone (3 µM) and β-FNA (0.4 µM) blocked significantly (p<0.0001) morphine- and DAMGO-induced depolarization of ∆ψ_m_ (Figure 5C). Pretreatment with naloxone (3 µM) and β-FNA (0.4 µM) alone significantly increased (polarization) ∆ψ_m_ (Figure 5C). Activation of TPCs with NAADP-AM (20 µM) significantly (p<0.0001) depolarized ∆ψ_m_ and the TPC antagonist NED-19 significantly (p<0.0001) blocked NAADP-, morphine- and DAMGO-induced decreases in ∆ψ_m_ (Figure 5D).

**Figure 6:**
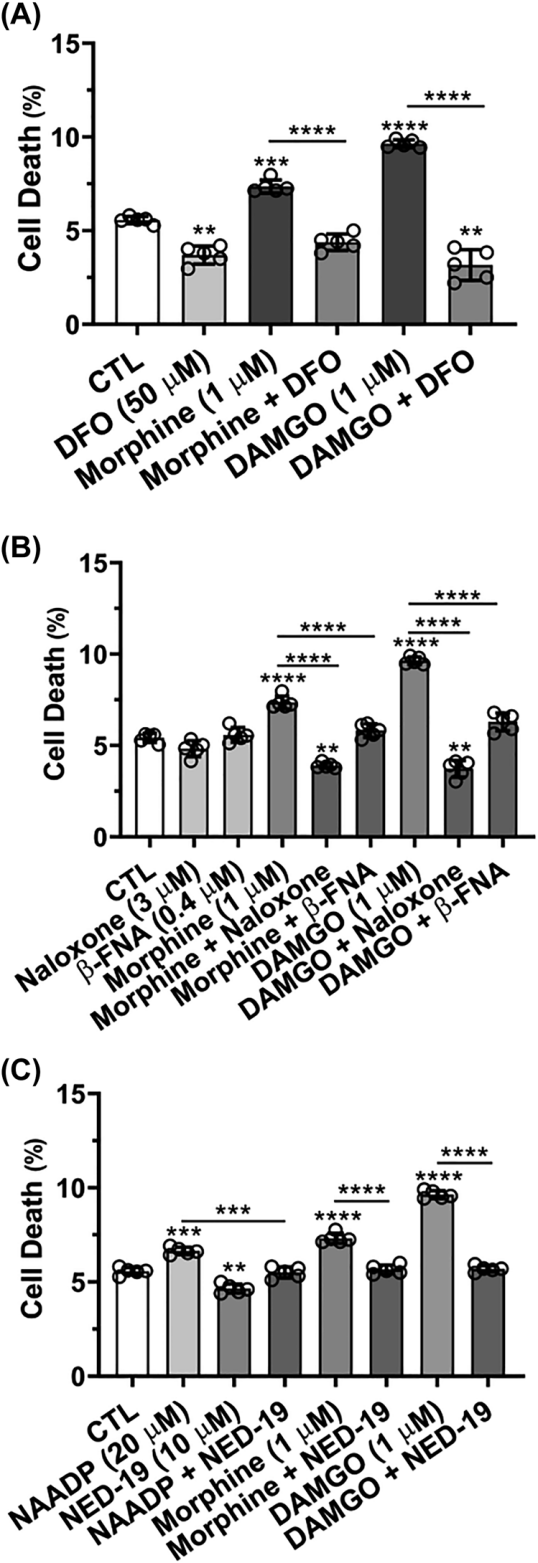
Effects of MOR agonists and antagonists, deferoxamine and two-pore channel activator and blocker on cell death. Propidium iodide was used to measure cell death of SH-SY5Y cells after 24 h drug treatments. (A) Cell death was significantly increased by morphine (1 µM) and DAMGO (1 µM); 30 min pre-treatment with DFO (50 µM) decreased cell death and blocked morphine- and DAMGO-induced cell death. (B) Morphine- and DAMGO-induced cell death was blocked by 30 min pre-treatments with naloxone (3.0 µM) or β-FNA (0.4 µM). (C) NAADP-AM- (20 µM), morphine- (1 µM) and DAMGO- (1 µM) induced increases in cell death were blocked by 30 min pre-treatments with NED-19 (10 µM); NED-19 alone significantly decreased levels of cell death. An ANOVA with Tukey’s post hoc multiple comparisons test was used for analysis. Each data point represents the mean fluorescence from 10,000 cells performed independently from five cell-culture preparations for each group (*n*=50,000). *n*=total number of cells for each group plotted. 50,000 cells were used for experimentation per group and no cells were intentionally excluded. (A) F(5, 24)=139.9, p<0.0001; (B) F(8, 36)=125.2, p<0.0001; (C) F(7, 32)=236.5, p<0.0001.

**Morphine- and DAMGO-induced cell death was blocked by DFO, MOR antagonists, and the TPC blocker NED-19:** Cell viability using propidium iodide was determined 24 h after addition of drug treatments. Cells pre-treated for 1 h with DFO (50 µM) significantly (p<0.01) decreased cell death and significantly (p<0.0001) blocked morphine- and DAMGO-induced cell death (Figure 6A). Pre-treatment with the MOR antagonists naloxone (3 µM) and β-FNA (0.4 µM) blocked significantly (p<0.0001) morphine- and DAMGO-induced cell death (Figure 6B). Naloxone (1 or 3 µM) and β-FNA (0.1 or 0.4 µM) alone had no significant effect compared to controls (Figure 6B and Supplemental Figure 2). Treatment of cells with NAADP-AM (20 µM) significantly (p<0.001) increased cell death and NED-19 blocked significantly NAADP- (p<0.001), morphine- (p<0.0001) and DAMGO- (p<0.0001) induced increases in cell death (Figure 6C). Cell death was determined to be mainly by apoptosis although some increases in lipid peroxidation (ferroptosis) were observed; necrosis was not apparent (Supplemental Figures 6 and 7).

**Figure 7:**
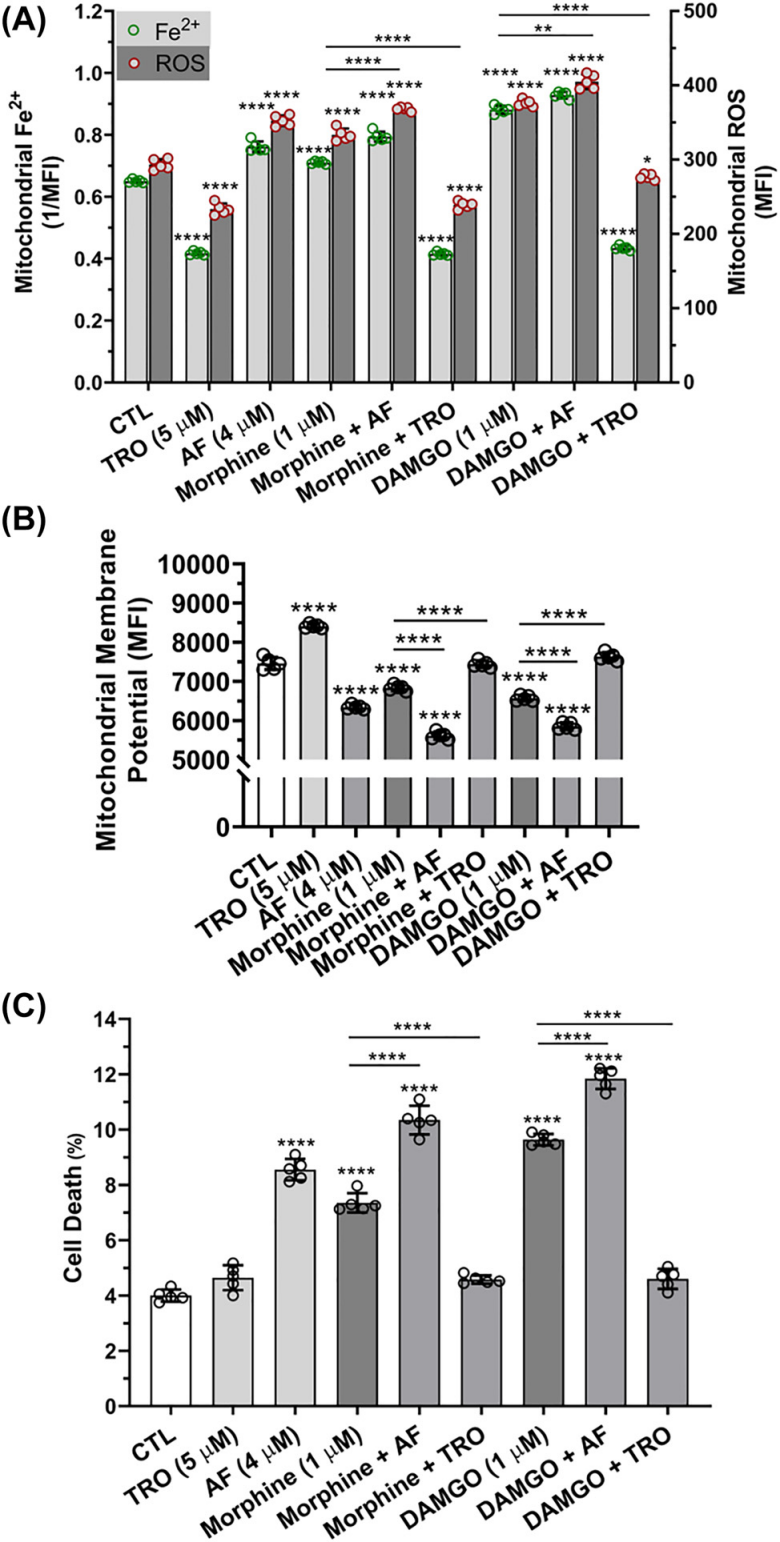
Effects of mitochondrial permeability transition pore activation and inhibition on MOR agonist-induced changes to mitochondrial iron, ROS and membrane potential as well as cell death. (A) MOR agonists morphine (1 µM) and DAMGO (1 µM) significantly increased levels of mitochondrial Fe^2+^ and ROS. Pre-treatment of cells with the mitochondrial permeability transition pore activator auranofin (AF, 4 µM) significantly increased morphine- and DAMGO-induced increases in levels of mitochondrial Fe^2+^ and ROS. Cells pre-treated with the mPTP inhibitor TRO (3 µM) blocked morphine- and DAMGO-induced increases in mitochondrial Fe^2+^ and ROS. (B) MOR agonists morphine (1 µM) and DAMGO (1 µM) significantly decreased levels of mitochondrial membrane potential. Pre-treatment of cells with the mitochondrial permeability transition pore activator auranofin (AF, 4 µM) significantly potentiated morphine- and DAMGO-induced decreases in mitochondrial membrane potential. Cells pre-treated with TRO (3 µM) blocked morphine- and DAMGO-induced decreases in mitochondrial membrane potential. (C) MOR agonists morphine (1 µM) and DAMGO (1 µM) increased cell death. Pre-treatment of cells with the mitochondrial permeability transition pore activator auranofin (AF, 4 µM) significantly potentiated morphine- and DAMGO-induced cell death. Cells pre-treated with TRO (3 µM) blocked morphine- and DAMGO-induced cell death. An ANOVA with Tukey’s post hoc multiple comparisons test was used for analysis. Each data point represents the mean fluorescence from 10,000 cells performed independently from five cell-culture preparations for each group (*n*=50,000). *n*=total number of cells for each group plotted. 50,000 cells were used for experimentation per group and no cells were intentionally excluded. (A) F(8, 72)=434.8, p<0.0001; (B) F(8, 36)=p<0.0001; (C) F(8, 36)=346.9, p<0.0001.

**Effects of mitochondrial permeability transition pore activation and inhibition on MOR agonist-induced changes to levels of mitochondrial iron, mitochondrial ROS, mitochondrial membrane potential, and cell death:** MOR agonists morphine (1 µM) and DAMGO (1 µM) significantly (p<0.0001) increased levels of mitochondrial Fe^2+^ and ROS (Figure 7A). Pre-treatment of cells with the mitochondrial permeability transition pore (mPTP) activator auranofin (AF, 4 µM) significantly (p<0.0001) increased morphine- and DAMGO-induced increases in levels of mitochondrial Fe^2+^ and ROS (Figure 7A). Cells pre-treated with the mPTP inhibitor TRO-19622 (TRO, 3 µM) significantly blocked morphine- and DAMGO-induced increases in mitochondrial Fe^2+^ and ROS (Figure 7A). MOR agonists morphine (1 µM) and DAMGO (1 µM) significantly (p<0.0001) decreased levels of mitochondrial membrane potential (Figure 7B). Pre-treatment of cells with auranofin (AF, 4 µM) significantly (p<0.0001) potentiated morphine- and DAMGO-induced decreases in ∆ψ_m_, and pre-treatment with TRO-19622 (TRO, 3 µM) significantly (p<0.0001) blocked morphine- and DAMGO-induced decreases in ∆ψ_m_ (Figure 7B). MOR agonists morphine (1 µM) and DAMGO (1 µM) significantly (p<0.0001) increased cell death (Figure 7C). Pre-treatment of cells with auranofin (AF, 4 µM) significantly (p<0.0001) potentiated morphine- and DAMGO-induced cell death while pre-treatment of cells with TRO-19622 (TRO, 3 µM) significantly (p<0.0001) blocked morphine- and DAMGO-induced cell death (Figure 7C).

## Discussion

**Figure 8:**
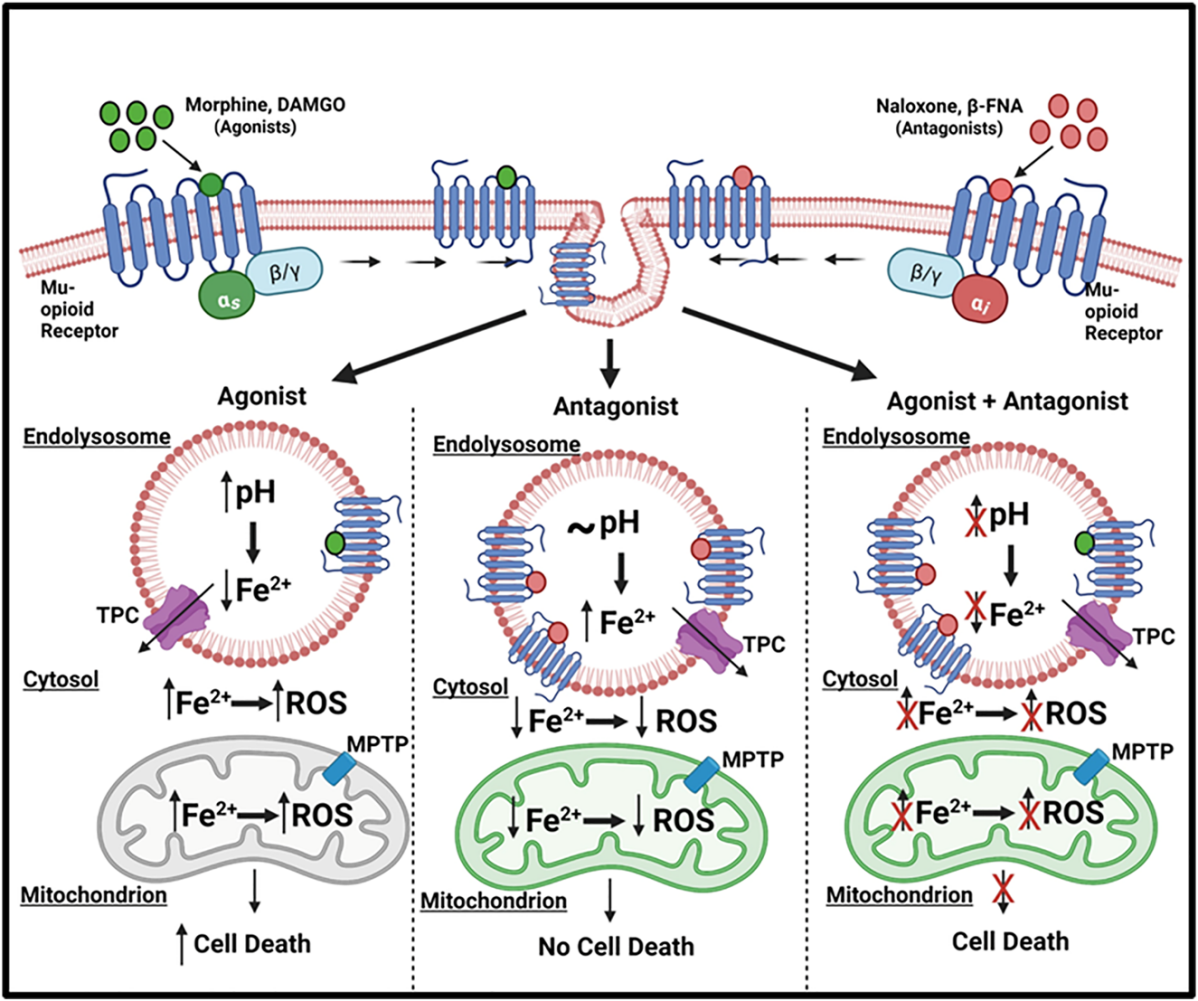
MOR activation-induced de-acidification of endolysosomes caused ferrous iron to be released from endolysosomes and this released iron increased levels of cytosolic and mitochondrial iron and ROS as well as cell death: MOR agonists morphine and DAMGO increased endolysosome pH and this de-acidification induced efflux of ferrous iron from endolysosomes through two-pore channels. The released iron was sufficient to increase iron levels in the cytosol and in mitochondria. The effects on mitochondrial iron and ROS levels appear to involve mitochondrial permeability transition pores. Decreased levels of ferrous iron in endolysosomes and increased levels of ferrous iron in cytosol and mitochondria led to increased cell death. The MOR antagonists naloxone and β-FNA blocked these MOR agonist-induced effects. Naloxone (3 µM) or β-FNA (0.4 µM) alone increased endolysosome iron resulting in decreased levels of cytosolic and mitochondrial iron as well as ROS.

Opioids are widely used clinically as analgesics and are prone to abuse due to their euphoric and dependency effects; their therapeutic and adverse effects are mediated by MORs [1, 2, 47]. Opioid activation of MORs increases ROS levels and induces cell death [5, 7, 9, 48]. However, little is known about the extent to which and mechanisms by which readily-releasable stores of endolysosome iron are sufficient to cause MOR-mediated increases in ROS levels and cell death. Mechanistically, we found that MOR agonists de-acidified endolysosomes, decreased Fe^2+^ levels in endolysosomes, increased cytosolic and mitochondrial Fe^2+^ and ROS levels, depolarized mitochondrial membrane potentials, and induced cell death; effects blocked by MOR antagonists, endolysosome iron chelation, an antagonist of endolysosome-resident TPCs, and an antagonist of mPTPs (Figure 8). Thus, opioid agonist-induced increases in cytosolic and mitochondrial Fe^2+^ and ROS as well as cell death appear to be downstream of endolysosome de-acidification and subsequent release of Fe^2+^ from endolysosomes.

Endolysosomes regulate numerous physiological functions and are involved in the pathogenesis of many diseases [49, 50]. Containing high levels of transition metals including iron, endolysosomes are central to iron trafficking and redox signaling; inhibition of endolysosome acidity triggers iron dysregulation [28, 29, 32, 33]. Because Fe^2+^ generates ROS via Fenton-like chemistry, it was important to determine the extent to which and mechanisms by which opioid agonist-induced effects on endolysosome pH and iron affected downstream events including ROS, mitochondria dysfunction, and cell death.

MORs are G-protein-coupled receptors (GPCRs) and their activation by opioid-agonists inhibits G-protein-mediated intracellular effectors including adenylate cyclase (AC) and decreases cAMP levels [51–53]. Intracellularly, cAMP regulates endolysosome acidification; in the absence of AC, endolysosomes fail to acidify (Supplementary Figure 8) [54, 55]. Notably, morphine and DAMGO stimulate [^35^S]GTPγS binding to G proteins and inhibit AC; actions blocked by the MOR antagonists naloxone and β-FNA [56, 57] as well as pertussis toxin [53]. Although not investigated here, these mechanisms may help explain our current findings because we previously demonstrated in primary cortical neurons and U87MG astrocytoma cells that morphine-induced endolysosome de-acidification, as well as increases in the cytoplasmic protein levels of ferritin heavy chain, were mediated through MORs and Gαi-protein signaling [35].

MORs are functionally active on the cell surface but have been shown to be active once endocytosed [4]. The opioid agonist morphine and the opioid antagonist naloxone are cell membrane-permeant and affect endosome-resident MORs whereas the MOR agonist DAMGO and the MOR antagonist β-FNA appear to function only through cell surface MORs [4, 58]. Because of those findings and the finding that DAMGO induces MOR endocytosis [59, 60], we cannot exclude the possible involvement of endosome-resident MORs in our results.

The morphine- and DAMGO-induced efflux of endolysosome Fe^2+^ was necessary and sufficient to increase cytosolic and mitochondrial Fe^2+^ levels because these effects were blocked by the endocytosed iron chelator DFO. Moreover, naloxone and β-FNA blocked the morphine- and DAMGO-induced efflux of endolysosome Fe^2+^ and the increases in cytosolic and mitochondrial Fe^2+^, which further supports the opioid agonist-induced subcellular effects on Fe^2+^ levels. Additionally, decreased mitochondrial iron levels induce mitochondrial swelling [61, 62] and we observed, qualitatively, opioid antagonist-induced mitochondrial swelling likely due to decreased mitochondrial Fe^2+^ levels.

Agreeing with previous studies demonstrating morphine “reduced the number of acidic vesicular organelles” (increased endolysosome pH) [35, 63], we found that morphine- and DAMGO-induced endolysosome de-acidification was blocked by naloxone and β-FNA. Endolysosome de-acidification induces an efflux of divalent cations through endolysosome-resident channels [42, 44]. We focused on endolysosome-resident TPCs because activation by the endogenous agonist NAADP increased the efflux of divalent cations from endolysosomes [30, 46]. Here, we confirmed that NAADP de-acidified endolysosomes [64], decreased endolysosome Fe^2+^ levels, and increased cytosolic and mitochondrial Fe^2+^ levels. The TPC antagonist NED-19 blocked what appears to be a constitutive leak of endolysosome Fe^2+^ and also blocked NAADP-, morphine-, and DAMGO-induced efflux of endolysosomes Fe^2+^ as well as the increases in cytosolic and mitochondrial Fe^2+^. Thus, NAADP-, morphine- and DAMGO-induced increases in cytosolic and mitochondrial Fe^2+^ appear to be downstream of their effects on endolysosome stores of Fe^2+^.

Opioid agonists increase ROS levels by decreasing antioxidant levels including catalase and glutathione peroxidase that remove excess hydrogen peroxide (H_2_O_2_) and by Fenton-like chemistry [65–68]. Following our findings that opioid agonists increased cytosolic and mitochondrial Fe^2+^ levels, it was important to determine if endolysosome Fe^2+^ stores were necessary and sufficient to increase cytosolic and mitochondrial ROS levels. Morphine and DAMGO increased cytosolic and mitochondrial ROS levels, which are consistent with previous findings [69, 70]; DFO, MOR antagonists and NED-19 blocked these effects, which strongly suggests the involvement of endolysosome Fe^2+^, MORs, and TPCs. Thus, opioid-induced increases in ROS levels appear to be downstream of MOR-mediated release of endolysosome iron.

Increases in ROS induce oxidative damage and thereby influence the development of opioid dependency [71, 72]. Central to oxidative damage and cell death is apoptosis via mitochondrial dysfunction [12]. Depolarization of ∆ψ_m_ is an early mitochondrial event leading to cell death [16] that can result from opening of the mPTP [18], and opioids have been shown to depolarize the ∆ψ_m_ [22]. Morphine and DAMGO both depolarized ∆ψ_m_; events blocked by DFO, naloxone and β-FNA, and NED-19. Moreover, the mPTP activator auranofin significantly enhanced morphine- and DAMGO-induced ∆ψ_m_ depolarization; events blocked by the mPTP inhibitor TRO. Thus, endolysosome iron appears to play an important role in maintaining the mitochondria membrane potential as well as endolysosome-to-mitochondrial signaling.

Because morphine and DAMGO depolarized the ∆ψ_m_, we next determined effects of opioids on cell death. Confirming previous findings [9, 48, 73], we found that morphine and DAMGO induced cell death. However, some studies suggest otherwise. Morphine was reported to protect against compound-induced toxicity and that morphine alone did not induce cell death even at a very concentration of 200 µM [74, 75]. However, several issues might explain these differences in results. First, some of the above studies used the MTT assay for determining cell death; a method that is not particularly sensitive in detecting cellular toxicity [76]. Second, using the more sensitive PI staining for determining levels of cell death morphine even at high concentrations did not induce cell death [77, 78]. However, in those studies they washed the cells post-treatment prior to removing attached cells for analysis; this may have biased their results. Here, we showed that morphine- and DAMGO-induced cell death was blocked by DFO, naloxone, β-FNA, NED-19, and TRO, but was enhanced by auranofin. These findings confirm previous results demonstrating naloxone blocked morphine- and DAMGO-induced cell death [8]. Taken together, these data demonstrate multiple mechanisms involving MOR-induced cell death including receptor-mediated, endolysosome iron translocation to mitochondria, TPCs, mPTPs, and ∆ψ_m_.

Opioids are well-known to increase ROS levels, which is linked to opioid dependency and MOR desensitization [79, 80]. In addition to MOR antagonists, we found that DFO and the antagonists of TPCs and mPTPs inhibited opioid-induced increases in ROS levels, thereby, providing several mechanisms for potential therapeutic targets. However, it is important to acknowledge some limitations of this study. While we showed similar effects of two different opioid agonists, morphine and DAMGO, it remains possible that not all opioid agonists would act similarly. However, previous studies have demonstrated that methadone and codeine induce increases in ROS levels [81, 82]. Furthermore, our investigations were limited to the study of endolysosomes, mitochondria and the cytosol. Thus, we cannot rule out the potential involvement of other organelles. Moreover, our study was limited to the analysis of endolysosome Fe^2+^ and did not address the possible involvement of other divalent cations known to be contained in endolysosomes and that are known to be involved in the generation of ROS and in cell death.

In addition to blocking opioid agonists, the effects of MOR antagonists naloxone (3 µM) and β-FNA (0.4 µM) alone were somewhat surprising in that they caused Fe^2+^ retention in endolysosomes and inhibited what appears to be a constitutive leak of Fe^2+^ from endolysosomes. This is supported by similar effects observed with the endolysosome iron chelator DFO. Concentration-response relationships for naloxone and β-FNA were conducted and at low concentrations naloxone (1 µM) and β-FNA (0.1 µM) inhibited morphine and DAMGO effects by 40–50% but did not alter [Fe^2+^]_el_ (Supplemental Figures 2 and 4). However, at higher concentrations naloxone (3 µM) and β-FNA (0.4 µM) completely inhibited morphine- and DAMGO-induced effects and significantly increased [Fe^2+^]_el_ without inducing cell death (Figure 2 and Supplemental Figure 2). Correspondingly, many studies have observed MOR antagonists alone to be beneficial [83–85].

Naloxone and β-FNA have anti-inflammatory effects, suppress superoxide production, and can be neuroprotective [83–87]. Increased iron correlates with markers of chronic inflammation [88], has been implicated in the pathogenesis of sporadic neurodegenerative diseases [89], and DFO protects against neuroinflammation [90]. Therapeutically, chelating endolysosome iron with DFO improves learning and memory through an increase in antioxidants [91], attenuates memory impairment [92], and decreases synaptic loss, oxidative stress markers, and neuronal loss [93–95].

Overall, our study demonstrates that opioids induce an MOR-mediated efflux of endolysosome Fe^2+^ that is sufficient to increase cytosolic and mitochondrial Fe^2+^ levels, as well as increase oxidative stress and cell death. Thus, inter-organellar signaling of Fe^2+^ may be key to better understanding opioid pharmacotherapeutics and adverse effects of opioid analgesics.

## Supplementary Material

Supplementary Material Details
