# Supplementary material for: Mu opioid receptor-mediated release of endolysosome iron increases levels of mitochondrial iron, reactive oxygen species, and cell death
Source: NeuroImmune Pharm Ther. 2022 Sep 14;2(1):19–35. doi: 10.1515/nipt-2022-0013 (PMC10070011; doi:10.1515/nipt-2022-0013)
Supplement: Supplementary file 1 — Supplementary Material Details [file j_nipt-2022-0013_suppl.pdf]

Supplementary Materials

Mu opioid receptor-mediated release of endolysosome iron increases levels of mitochondrial iron, reactive oxygen species, and cell death

Peter W. Halcrow*[1], Nirmal Kumar*[1], Emily Hao[1], Nabab Khan[1], Olimpia Meucci[2] and Jonathan D. Geiger[1]**

Affiliations:

[1] Department of Biomedical Sciences, University of North Dakota School of Medicine and Health Sciences, Grand Forks, North Dakota

[2] Department of Physiology and Pharmacology, Drexel University School of Medicine, Philadelphia, Pennsylvania

Address Correspondence to:

Jonathan D. Geiger, Ph.D.
Chester Fritz Distinguished Professor
Department of Biomedical Sciences
University of North Dakota School of Medicine and Health Sciences
504 Hamline Street, Room 110
Grand Forks, North Dakota 58203
(701) 777-2183 (P); jonathan.geiger@und.edu

***These authors contributed equally to this work**

Supplemental Figure 1

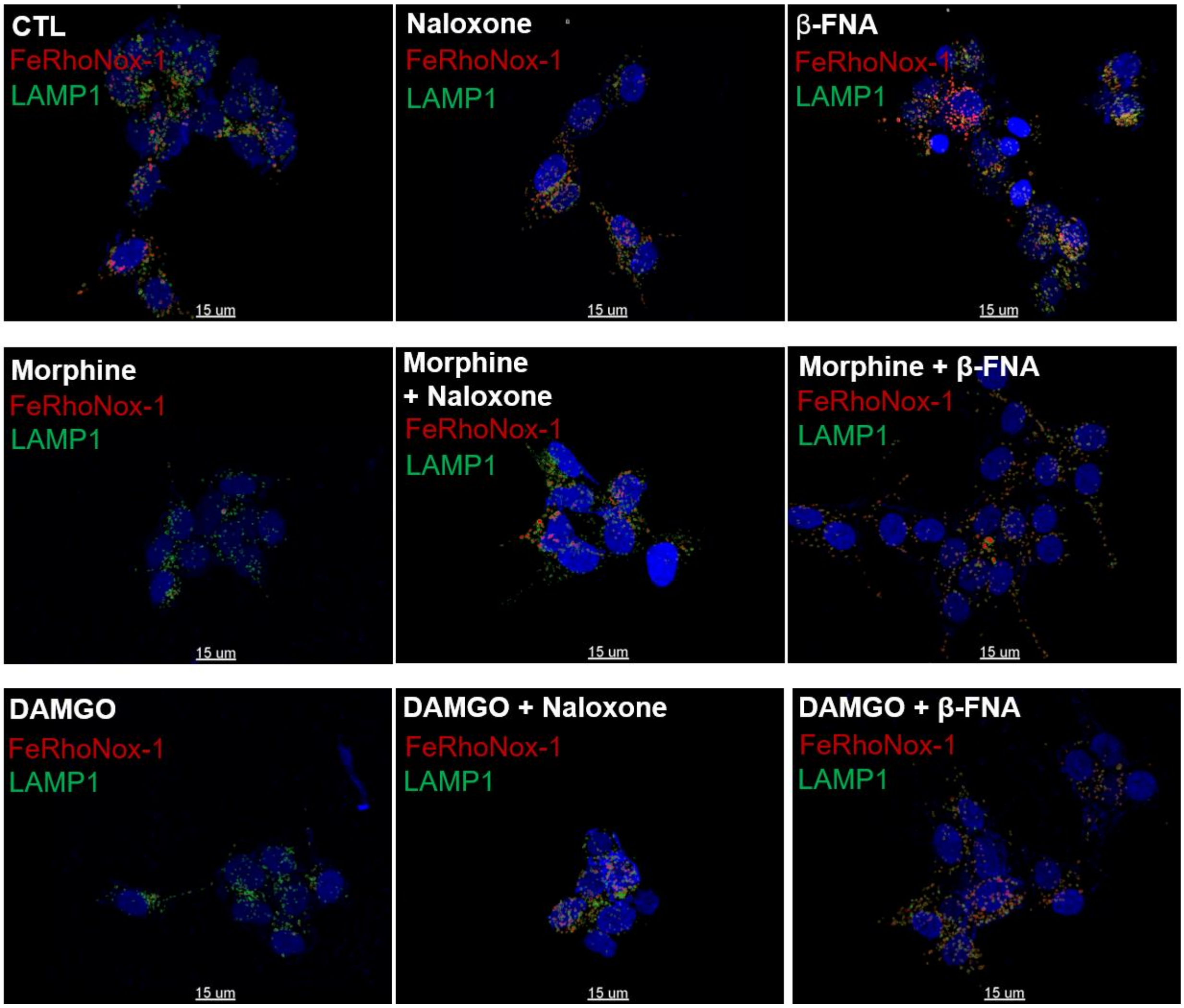

Figure 1: Morphine- and DAMGO-induced decreases in levels of endolysosome Fe^{2+} were blocked by the MOR antagonists naloxone and β-FNA. Qualitatively, the MOR agonists morphine and DAMGO decreased FeRhoNox-1 staining of endolysosome Fe^{2+} and these decreases were blocked by the MOR antagonists naloxone and β-FNA. Multiple-cell images were obtained using confocal scanning microscopy of SH-SY5Y cells treated for 30 min with water (CTL), morphine (1 μM), DAMGO (1 μM), naloxone (3 μM), and β-FNA (0.4 μM). Images were reconstructed using Imaris 3D software and

show FeRhoNox-1 staining (red) of Fe^{2+} inside of LAMP1-positive (green) endolysosomes. Quantitatively (Figure 2b), levels of endolysosome Fe^{2+} as indicated by mean fluorescence intensity (MFI) for FeRhoNox-1 staining were significantly ($p<0.0001$) decreased by morphine (1 µM) and DAMGO (1 µM); the decreases in FeRhoNox-1 staining were blocked by the MOR antagonists naloxone (3 µM) and β-FNA (0.4 µM).

Supplemental Figure 2

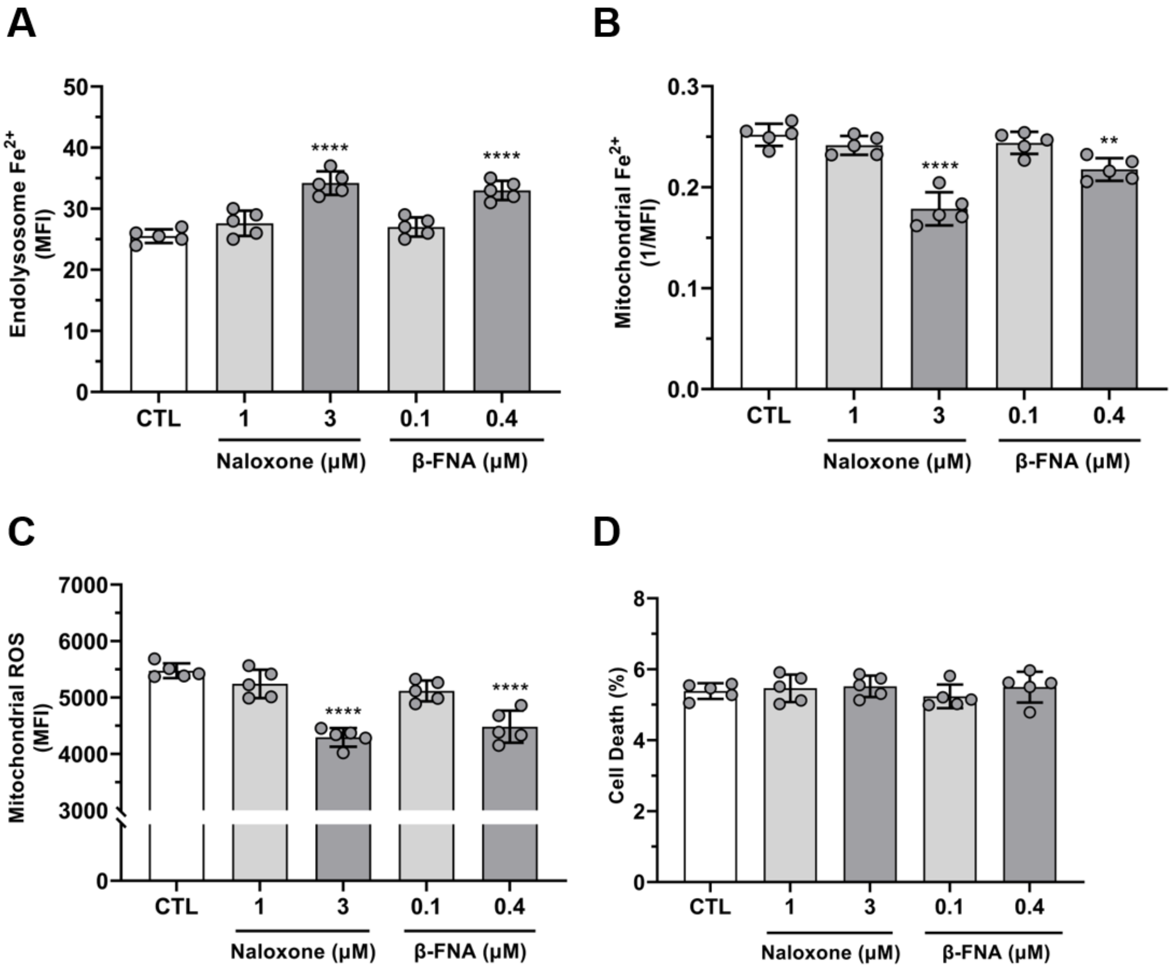

Figure 2: Concentration-response relationships for the MOR antagonists naloxone and β-FNA on levels of endolysosome Fe^{2+}, mitochondrial Fe^{2+} and ROS, and cell death. **(A)** Quantitatively, levels of endolysosome Fe^{2+} as indicated by mean fluorescence intensity (MFI) for FeRhoNox-1 staining were significantly ($p<0.0001$) increased by higher concentrations of MOR antagonists naloxone (3 μM) and β-FNA (0.4 μM). Each data point represents the mean fluorescence of 300 endolysosomes from ten cells performed independently in triplicate (n = 9,000). Cells were chosen randomly in the microscope's

field of view with 30 cells used for experimentation per group and no cells were intentionally excluded. An ANOVA with a Tukey's post hoc multiple comparisons test was used for analysis. (**B,C)** Mitochondrial iron levels ($[Fe^{2+}]_{mito}$) were determined by using the quenching dye rhodamine B-[(2,2'-bipyridine-4-yl)-aminocarbonyl]benzyl ester (RDA) and fluorescence data were expressed as 1/MFI. Levels of mitochondrial ROS ($[ROS]_{mito}$) were measured using MitoSox and data were expressed as MFI. At higher concentrations, the MOR antagonists naloxone (3 µM) and β-FNA (0.4 µM) significantly decreased levels of mitochondrial Fe^{2+} and ROS. **(D)** Propidium iodide was used to measure cell death of SH-SY5Y cells after 24 h drug treatments. No significant differences were observed for cell death at the lower and higher concentrations of the MOR antagonist naloxone (1 µM and 3 µM) and β-FNA (0.1 µM and 0.4 µM) for 24 h. An ANOVA with a Tukey's post hoc multiple comparisons test was used for analysis. Each data point represents the mean fluorescence from 10,000 cells performed independently five times for each group (n = 50,000). n = total number of cells for each group plotted. 50,000 cells were used for experimentation per group. (A) $F(4, 20) = 26.4$, $p < 0.0001$; (B) $F(4, 20) = 30.71$, $p < 0.0001$; (C) $F(4, 20) = 29.16$, $p < 0.0001$; (D) $F(4, 20) = 0.5586$, $p = 0.6953$

Supplemental Figure 3

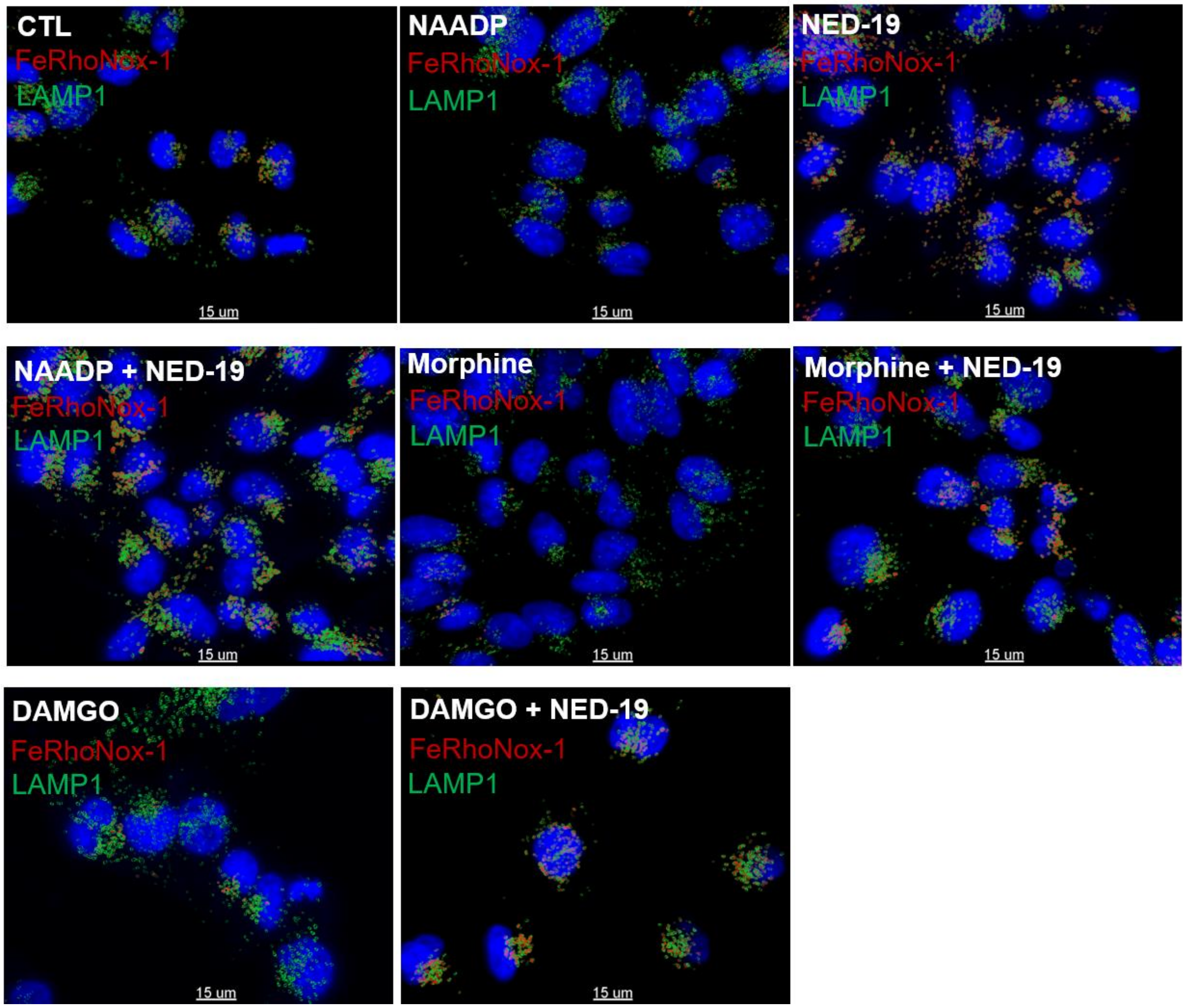

Figure 3: Morphine- and DAMGO-induced decreases in levels of endolysosome Fe^{2+} were blocked by NED-19, an inhibitor of two-pore channels. Qualitatively, activation of two-pore channels with the endogenous agonist NAADP-AM (20 µM) and the MOR agonists morphine (1 µM) and DAMGO (1 µM) decreased FeRhoNox-1 staining of endolysosome Fe^{2+}. The effects of NAADP-AM, morphine and DAMGO were blocked by the two-pore channel blocker NED-19 (10 µM). Multiple-cell images were obtained using confocal scanning microscopy of SH-SY5Y cells treated for 30 min with water or

DMSO (CTL), morphine (1 µM), DAMGO (1 µM), NAADP-AM (20 µM) and NED-19 (10 µM). Images were reconstructed using Imaris 3D software and show FeRhoNox-1 staining (red) of Fe^{2+} inside of LAMP1-positive (green) endolysosomes. Quantitatively (Figure 3b), levels of endolysosome Fe^{2+} as indicated by mean fluorescence intensity (MFI) for FeRhoNox-1 staining were significantly ($p<0.0001$) decreased by morphine (1 µM), DAMGO (1 µM) and NAADP-AM (20 µM). The decreases in FeRhoNox-1 staining were blocked by NED-19 (10 µM).

Supplemental Figure 4

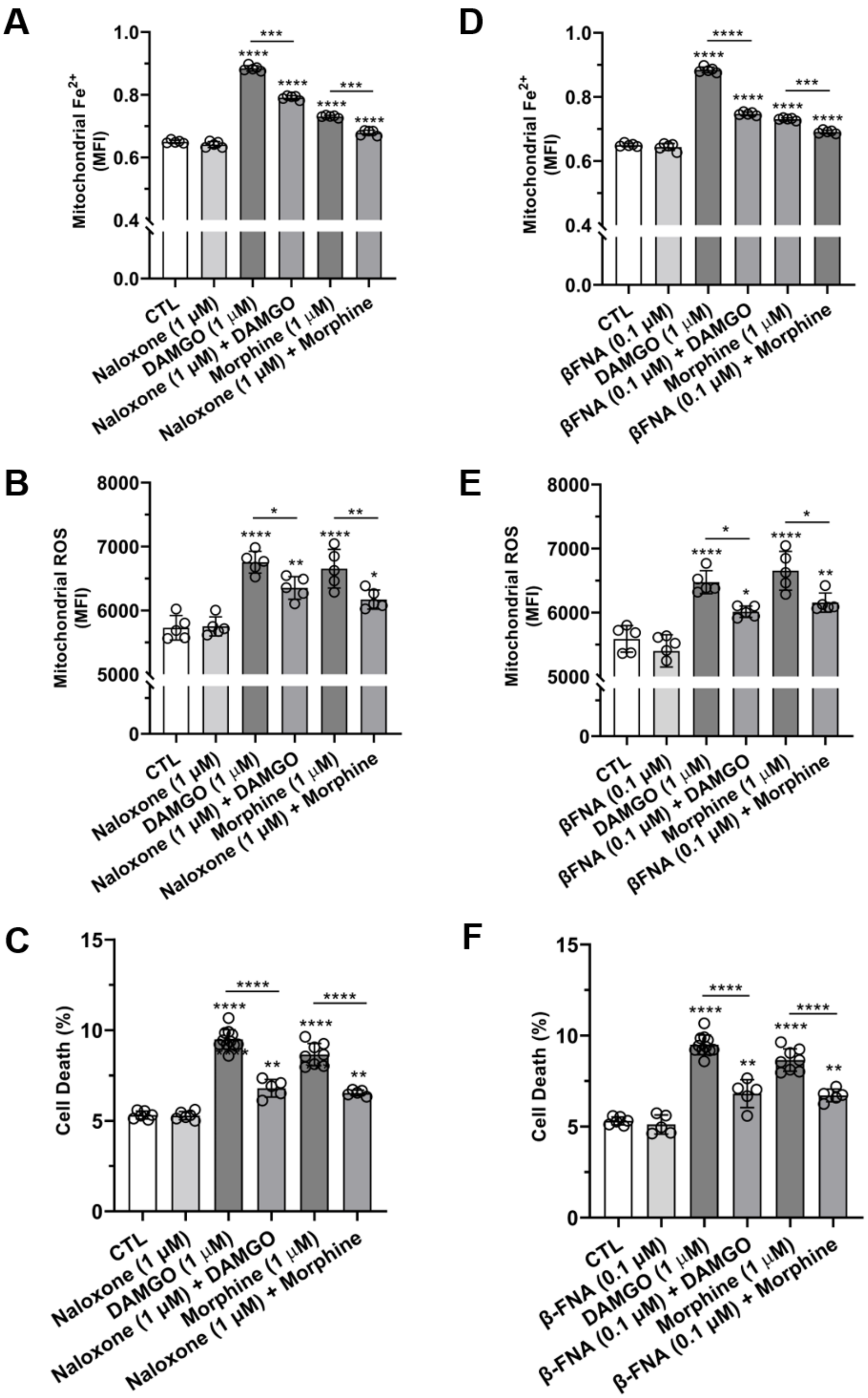

Figure 4: Effects of MOR agonists and antagonists on mitochondrial levels of Fe^{2+} and ROS as well as cell death. Mitochondrial iron levels ($[Fe^{2+}]_{mito}$) were determined by using the quenching dye rhodamine B-[(2,2'-bipyridine-4-yl)-aminocarbonyl]benzyl ester (RDA) and fluorescence data were expressed as 1/MFI. Levels of mitochondrial ROS ($[ROS]_{mito}$) were measured using MitoSox and data were expressed as MFI. **(A,B,D,E)** MOR agonists morphine (1 μM) and DAMGO (1 μM) significantly increased levels of mitochondrial Fe^{2+} and ROS; these increases were significantly decreased by the MOR antagonists naloxone (1 μM) and β-FNA (0.1 μM). (**C,F)** Propidium iodide was used to measure cell death of SH-SY5Y cells after 24 h treatments. Cell death was significantly increased by morphine (1 μM) and DAMGO (1 μM); 30 min pre-treatment with naloxone (1.0 μM) or β-FNA (0.1 μM) significantly decreased morphine- and DAMGO-induced cell death. An ANOVA with a Tukey's post hoc multiple comparisons test was used for analysis. Each data point represents the mean fluorescence from 10,000 cells performed independently five times for each group (n = 50,000). n = total number of cells for each group plotted. 50,000 cells were used for experimentation per group. (A) $F(5, 24) = 829.4$, $p < 0.0001$; (B) $F(5, 24) = 24.58$, $p < 0.0001$; (C) $F(5, 35) = 118.1$, $p < 0.0001$; (D) $F(5, 24) = 805.1$, $p < 0.0001$; (E) $F(5, 25) = 28.82$, $p < 0.0001$; (F) $F(5, 34) = 81.95$, $p < 0.0001$

Supplemental Figure 5

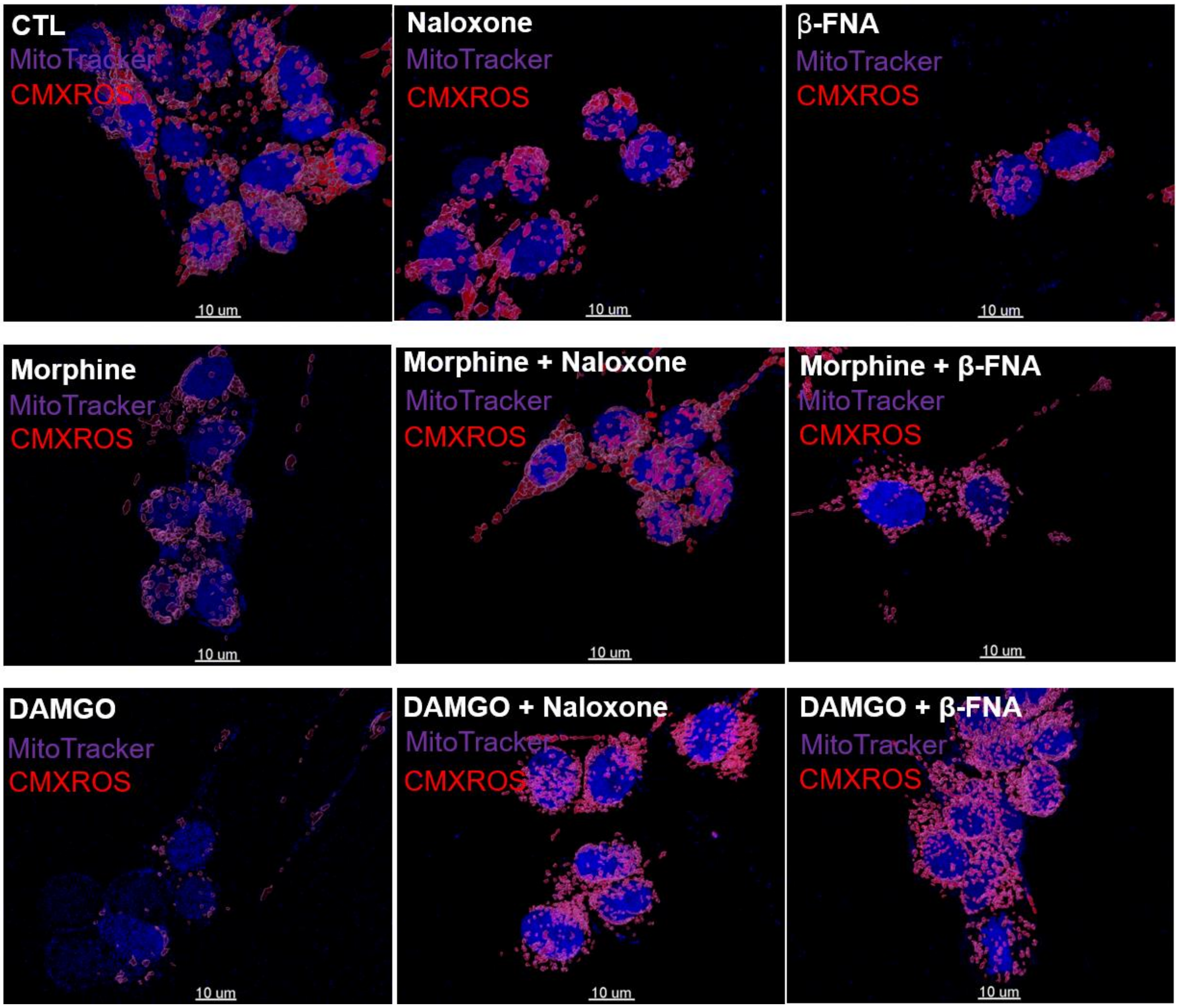

Figure 5: <u>Effects of MOR agonists and antagonists and two-pore channel activator and blocker on mitochondrial membrane potential.</u> Mitochondrial membrane potential ($\Delta\psi_m$) was measured using the dye CMXROS; dye intensity decreases with $\Delta\psi_m$ depolarization. Representative confocal scanning microscope with multiple-cell images of mitochondria in SH-SY5Y cells treated for 30 min with water (CTL), and the MOR agonists morphine (1 μM) and DAMGO (1 μM) in the absence or presence of MOR antagonists naloxone (3 μM) and β-FNA (0.4 μM). Images were reconstructed using

Imaris 3D software; illustrated are MitoTracker-(purple) positive mitochondria containing CMXROS stain (red). Qualitatively, treatment of cells for 30 min with morphine (1 µM) and DAMGO (1 µM) decreased CMXROS fluorescence, and naloxone and β-FNA inhibited morphine- and DAMGO-induced decreases in CMXROS fluorescence. Quantitatively (Figure 5c)**,** morphine (1 µM) and DAMGO (1 µM) significantly decreased CMXROS mean fluorescence intensity (depolarization); 30 min pre-treatment with naloxone (1.0 µM) or β-FNA (0.4 µM) followed by treatment with morphine (1 µM) or DAMGO (1 µM) resulted in significantly increased levels of CMXROS (polarization).

Supplemental Figure 6

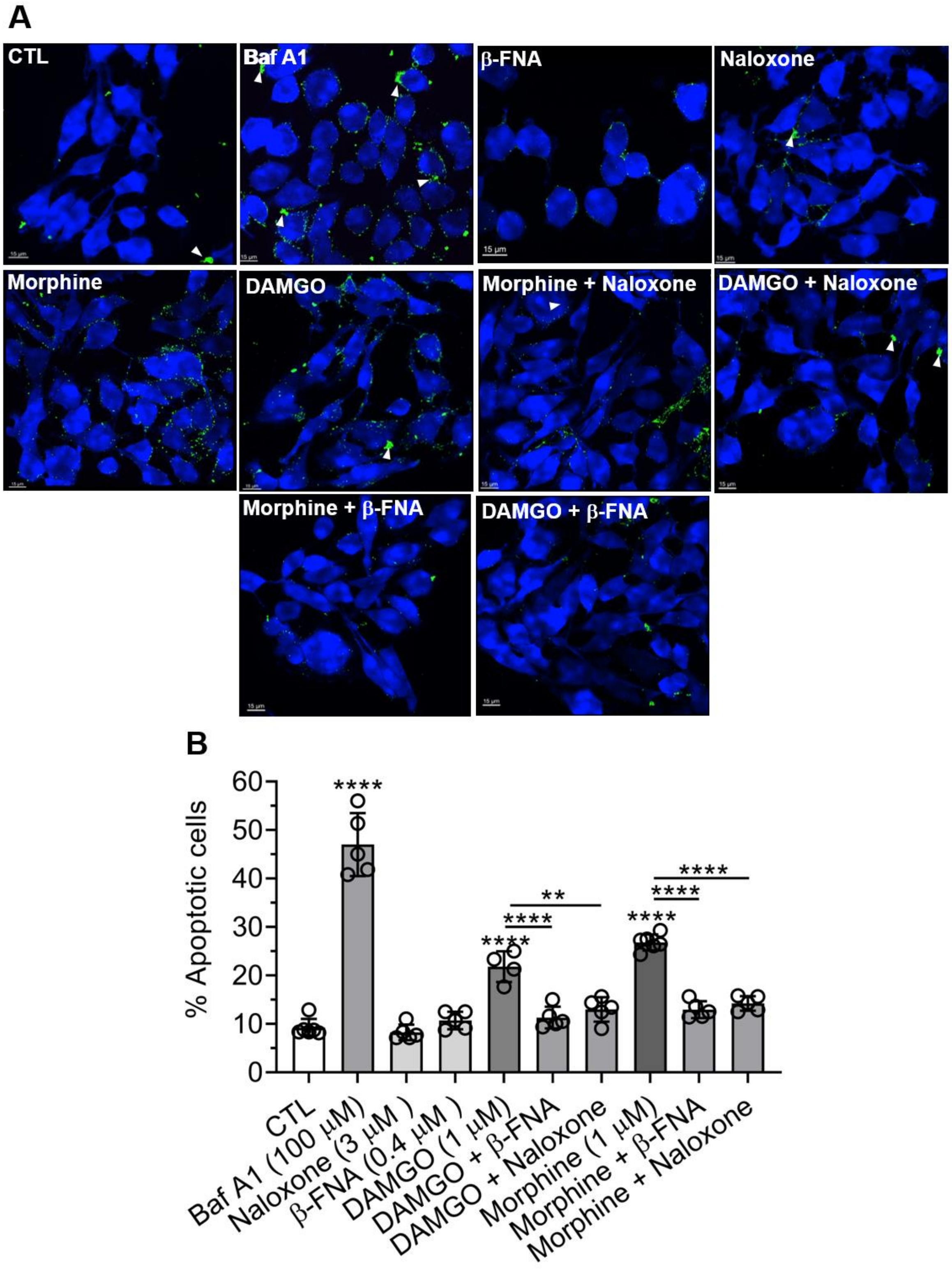

Figure 6: Morphine- and DAMGO-induced apoptosis was blocked by the MOR antagonists naloxone and β-FNA. **(A)** Qualitatively, the MOR agonists morphine (1 μM) and DAMGO (1 μM) increased Apopxin green staining (indication of apoptosis cells and the white arrowheads specify apoptotic body release) and these increases were blocked by the MOR antagonists naloxone (3 μM) and β-FNA (0.4 μM). Images were obtained using confocal scanning microscopy of SH-SY5Y cells treated for 24 h with water (CTL), morphine (1 μM), DAMGO (1 μM), naloxone (3 μM), and β-FNA (0.4 μM). Images were analyzed using Imaris software and show staining of Apopxin Green which labels phosphatidylserine (PS) on the cell surface of apoptotic cells (green fluorescence), and CytoCalcein Violet labels the cytoplasm of living cells (blue fluorescence). **(B)** Quantitatively, apoptotic cells as indicated by Apopxin green staining were significantly ($p<0.0001$) increased by morphine (1 μM) and DAMGO (1 μM); these increases in Apopxin green staining were blocked by the MOR antagonists naloxone (3 μM) and β-FNA (0.4 μM). An ANOVA with a Tukey's post hoc multiple comparisons test was used for analysis. Each data point represents the mean fluorescence of 30 cells performed independently five times for each group (n = 150). n = total number of cells for each group plotted. Cells were chosen randomly in the microscope's field of view and no cells were intentionally excluded. Scale bar = 15 μm. (B) $F(9, 41) = 92.71$, $p < 0.0001$

Supplemental Figure 7

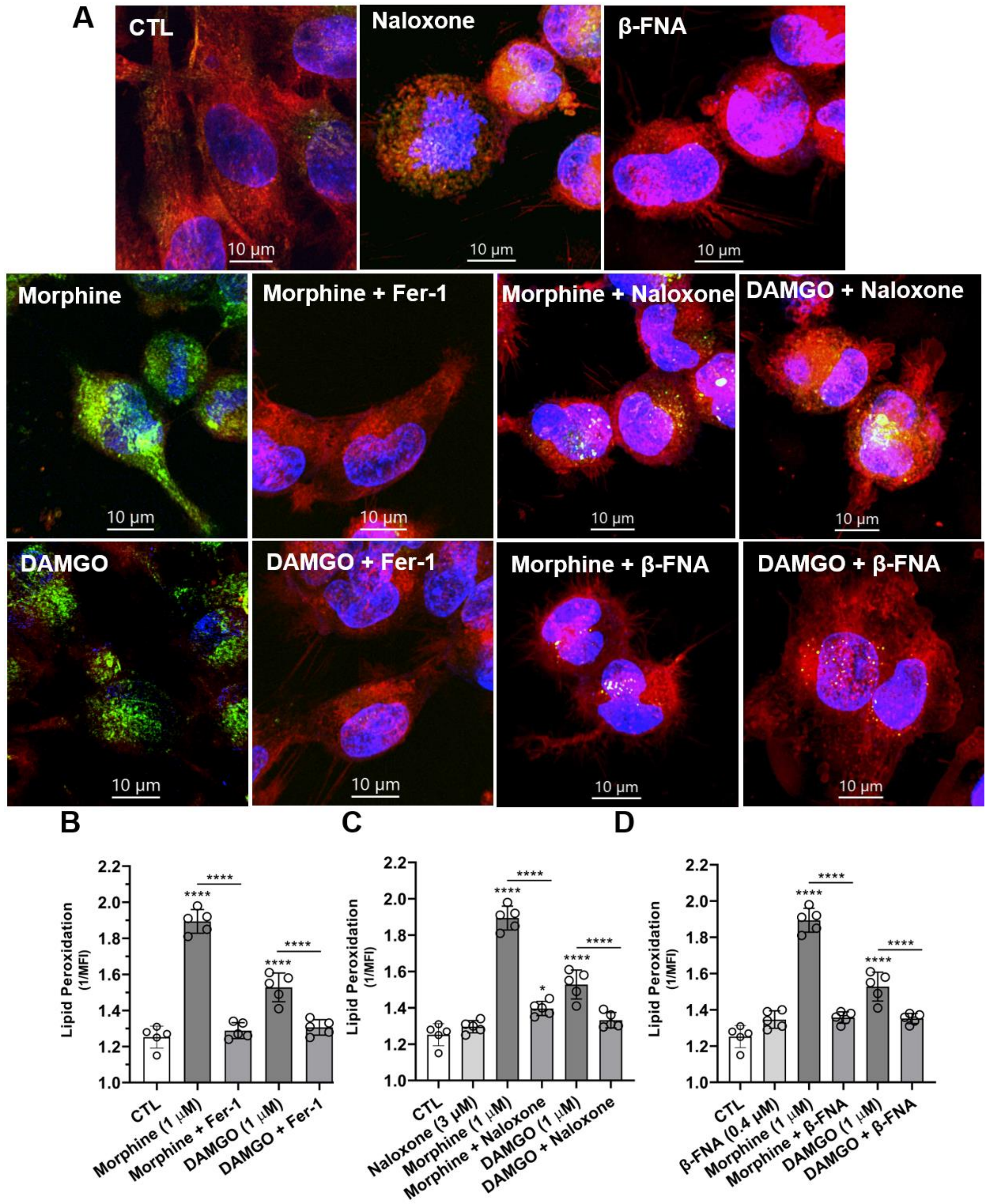

Figure 7: Morphine- and DAMGO-induced increases in lipid peroxidation were blocked by the MOR antagonists naloxone and β-FNA and also by the ferroptosis inhibitor ferrostatin-1. **(A)** Qualitatively, the MOR agonists morphine (1 µM) and DAMGO (1 µM) increased lipid peroxidation staining fluorescence (C11-BODIPY 581/591); these increases were blocked by the MOR antagonists naloxone (3 µM) and β-FNA (0.4 µM) as well as the ferroptosis inhibitor ferrostatin-1 (Fer-1, 5 µM). Images were obtained using confocal scanning microscopy of SH-SY5Y cells treated for 24 h with water or DMSO (CTL), morphine (1 µM), DAMGO (1 µM), naloxone (3 µM), β-FNA (0.4 µM) and Fer-1 (5 µM). Images were analyzed using Imaris software and show staining of C11-BODIPY 581/591 (red and green) and nucleus staining with Hoechst 33342 (blue). **(B)** Quantitatively, lipid peroxidation as indicated by changes in C11-BODIPY 581/591 fluorescence were significantly ($p<0.0001$) increased by morphine (1 µM) and DAMGO (1 µM); these increases in lipid peroxidation were blocked by the MOR antagonists naloxone (3 µM) and β-FNA (0.4 µM) as well as Fer-1 (5 µM). An ANOVA with a Tukey's post hoc multiple comparisons test was used for analysis. Each data point represents the mean fluorescence of 30 cells performed independently five times for each group ($n = 150$). n = total number of cells for each group plotted. Cells were chosen randomly in the microscope's field of view and no cells were intentionally excluded. Scale bar = 10 µm. (B) $F(4, 20) = 99.53$, $p < 0.0001$; (C) $F(5, 24) = 90.22$, $p < 0.0001$; (D) $F(5, 24) = 88.15$, $p < 0.0001$

Supplemental Figure 8

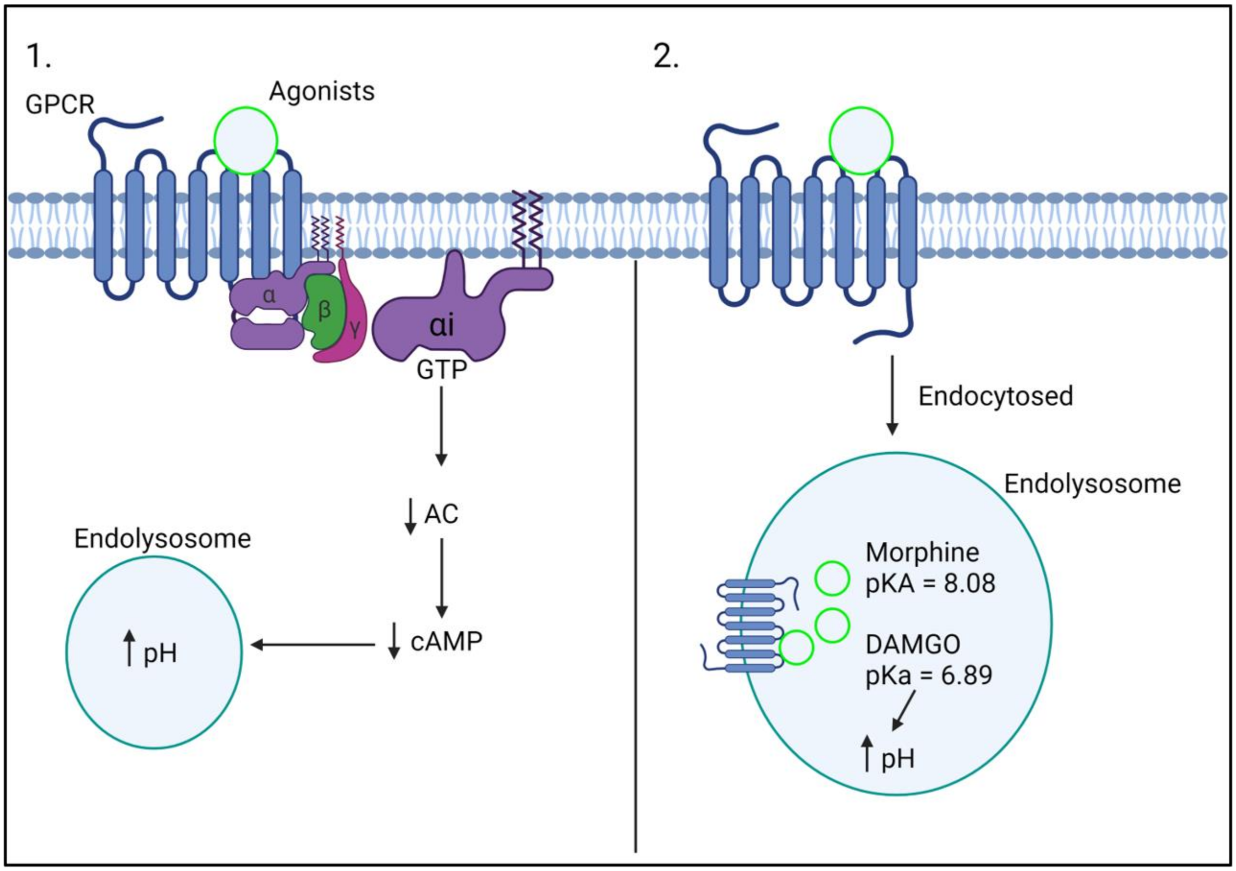

Figure 8: <u>Two potential mechanisms by which morphine and DAMGO de-acidify endolysosomes</u>. 1. Morphine and DAMGO bind GPCRs at the plasma membrane resulting in G-protein conformational changes and GTP binding to Gαi subunits. These changes in G-proteins by opioids are well-known to decrease expression and activity levels of adenylyl cyclase (AC) and levels of cyclic adenosine monophosphate (cAMP). Decreased levels of AC and cAMP have been linked to increases in endolysosome pH. 2. Morphine and DAMGO have pKa values of 8.08 and 6.89, respectively. Compounds with neutral to basic pKa values are known to increase the pH of the acidic lumen within endolysosomes.
